# Numerical investigation of the fractional diffusion wave equation with exponential kernel via cubic B-Spline approach

**DOI:** 10.1371/journal.pone.0295525

**Published:** 2023-12-15

**Authors:** Madiha Shafiq, Muhammad Abbas, Homan Emadifar, Ahmed SM Alzaidi, Tahir Nazir, Farah Aini Abdullah

**Affiliations:** 1 Department of Mathematics, University of Sargodha, Sargodha, Pakistan; 2 Department of Mathematics, Saveetha School of Engineering, Saveetha Institute of Medical and Technical Sciences, Chennai, Tamil Nadu, India; 3 Department of Mathematics, Hamedan Branch, Islamic Azad University, Hamedan, Iran; 4 Department of Mathematics and Statistics, College of Science, Taif University, Taif, Saudi Arabia; 5 School of Mathematical Sciences, Universiti Sains Malaysia, Penang, Malaysia; Qujing Normal University, CHINA

## Abstract

Splines are piecewise polynomials that are as smooth as they can be without forming a single polynomial. They are linked at specific points known as knots. Splines are useful for a variety of problems in numerical analysis and applied mathematics because they are simple to store and manipulate on a computer. These include, for example, numerical quadrature, function approximation, data fitting, etc. In this study, cubic B-spline (CBS) functions are used to numerically solve the time fractional diffusion wave equation (TFDWE) with Caputo-Fabrizio derivative. To discretize the spatial and temporal derivatives, CBS with *θ*-weighted scheme and the finite difference approach are utilized, respectively. Convergence analysis and stability of the presented method are analyzed. Some examples are used to validate the suggested scheme, and they show that it is feasible and fairly accurate.

## 1 Introduction

The area of mathematical analysis known as fractional calculus (FC) studies the applications of non-integer order integrals and derivatives. FC has generated significant interest in past decades. An extensive study on this topic is discussed in [1, 2]. Because of its wide uses in the numerous branches of engineering and science, the field of FC is highly regarded and significant. For instance, the FC has been implemented in the control theory [3], viscoelastic and viscoplastic flow [4], continuum mechanics [5], tumor development [6], transport problems [7], random walks [8], turbulence [9, 10], coronavirus [11, 12] and dynamical systems [13, 14]. Multitudinous authors [15–19] have called attention to the fractional integrals and derivatives that are certainly suitable for demonstrating memory and hereditary characteristics of several materials and methodologies that are handled by anomalous diffusion. In many cases, fractional problems characterised by fractional partial differential equations (FPDEs) behave more appropriately than their integer order equivalents. In general, it is impossible to obtain the exact result of the maximum of the FPDEs. As a result, many contemporary research articles focus on the quest for numerical approaches. In this paper, the following TFDWE with damping and reaction terms for different numerical outcomes shall be studied:

$$\begin{array}{c} \frac{\partial^{\gamma }w\left(u,t\right)}{\partial t^{\gamma }}+\delta \frac{\partial w(u,t)}{\partial t}+\sigma w\left(u,t\right)-\frac{\partial^{2}w\left(u,t\right)}{\partial u^{2}}=q\left(u,t\right),\;\;\gamma \in \left(1,2\right),\;\;u\in \left[a,b\right],\;\;t\in \left[t_{0},T\right], \end{array}$$ (1)

with initial conditions (ICs):

$$\begin{array}{c} w\left(u,t_{0}\right)=\psi_{1}\left(u\right),\;\;\;\;w_{t}\left(u,t_{0}\right)=\psi_{2}\left(u\right) \end{array}$$ (2)

and boundary conditions (BCs):

$$\begin{array}{c} w\left(a,t\right)=\phi_{1}\left(t\right),\;\;\;\;w\left(b,t\right)=\phi_{2}\left(t\right), \end{array}$$ (3)

where *δ* and *σ* are coefficients of the damping and reaction terms, respectively. *q*(*u*, *t*) is the source term and *w*_*t*_(*u*, *t*_0_) represents the derivative of the function *w*(*u*, *t*) with respect to time *t* at *t* = *t*_0_. The Caputo-Fabrizio fractional derivative (CFFD) $\frac{\partial^{\gamma }}{\partial t^{\gamma }}w\left(u,t\right)$ is described as:

$$\begin{array}{c} \frac{\partial^{\gamma }}{\partial t^{\gamma }}w\left(u,t\right)=\frac{R(\gamma )}{2-\gamma }\int_{0}^{t}\frac{\partial^{2}}{\partial v^{2}}w\left(u,v\right)\;\text{exp}\;[-\frac{\gamma }{2-\gamma }\left(t-v\right)]dv, \end{array}$$ (4)

where *R*(*γ*) is the normalization function fulfils *R*(0) = *R*(1) = 1. Recently, several articles have discussed the CFFD’s applications. For example, Cruz-Duarte *et al.* [20] propounded a closed formula for the Gaussian-based CFFD with signal processing applications. Shafiq *et al*. [21] demonstrated the numerical results of diffusion equation (DE) using spline functions. For solving optimal control problems, Mortezaee *et al*. [22] developed generalized fuzzy hyperbolic model. Zhou *et al.* [23] presented the model of solute transport and non-Darcian flow in porous media involving CFFD. The hepatitis E dynamics in CFFD sense has been presented by Khan *et al.* [24]. Rahman *et al.* [25] studied the coronavirus (COVID-19) mathematical model with CFFD. Alshabanat *et al.* [26] presented the generalization of CFFD and its applications on electrical circuits.

Recently, numerous numerical methodologies for resolving TFDWEs have been established. Ding and Li [27] developed two numerical techniques for solving the TFDWE with a reaction term. Avazzadeh *et al.* [28] used the radial basis functions to resolve the TFDWE. For fractional wave equation (FWE), Khader and Adel [29] introduced a Hermite formula based algorithm. Chatterjee *et al.* [30] proposed a method based on Bernstein polynomials for non-linear TFDWE. Zeng [31] proposed two schemes for the TFDWE. The Legendre wavelets based computational method has been presented by Hooshmandasl *et al.* [32] to resolve the fractional sub-diffusion and TFDWEs. Ali *et al.* [33] applied implicit difference technique for computational solution of TFDWE. Sweilam *et al.* [34] investigated a computational study for variable order nonlinear FWE. Zhou and Xu [35] used Chebyshev wavelets collocation scheme for TFDWE. More classical work on TFDWE can be found in [36–40].

Koleva and Vulkov [41] used Jumarie’s derivative to solve the Black-Scholes equation. Ganji *et al.* [42] proposed a new method for Klein-Gordon equation (KGE) with Caputo fractional derivative (CFD) through clique polynomials. Adomian decomposition technique has been utilized by Birajdar [43] for solving Navier-Stokes fractional equation. The numerical simulation for Fokker-Planck fractional equation has been presented by Mahdy [44]. Hosseini *et al.* [45] proposed singular boundary technique for fractional DE with CFD. Akgül *et al.* [46] presented an accurate method for Lane-Emden fractional type equations. Babu *et al.* [47] developed Localized Differential Quadrature scheme for solving viscous Burgers’ equation (BE).

Due to their simplicity, splines have been utilized by numerous researchers to resolve fractional differential equations. Wasim *et al.* [48] utilized hybrid B-spline scheme for solving Fisher and Huxley BEs. B-spline based computational methods have been proposed by Yaseen and Abbas [49, 50] for fractional Burgers’ and telegraph equations (TEs) with CFD. Akram *et al.* [51] presented extended CBS technique to solve non-linear TE involving CFD. Amin *et al.* [52] developed redefined extended CBS algorithm to resolve time-fractional KGE. Allen-Cahn equation by utilizing redefined CBS functions has been investigated by Khalid *et al.* [53] with CFD. Reaction-diffusion model with CFFD has been constructed by Akram *et al.* [54]. B-splines based technique for time-fractional DE with CFD has been presented by Yaseen *et al.* [55]. Akram *et al.* [56] developed a numerical approach which depends on extended CBS function for Fisher equation with CFD. Shafiq *et al*. [57] resolved the Burgers’ equations involving ABFD numerical using CBS. Kamran *et al.* [58] presented numerical simulation for BBM-BE with CFD using CBS functions. Shafiq *et al*. presented the CBS based algorithm to resolve the TFADE in the article [59].

The proposed attempt has been inspired by recent developments in the investigation of computational solutions to the TFDWE. As a result of its extensive use, our study’s objective is to apply CBS to the TFDWE. In the past, many researchers utilized B-spline techniques to resolve TFDWE, but no one has ever employed CBS approximations with CFFD. To solve the TFDWE, the CBS with *θ*-weighted technique is used. Analyses of the scheme’s convergence and stability are also carried out. By giving answers to a few numerical issues, the propounded approach’s efficacy and applicability are illustrated. We find that our proposed technique yields effective results by contrasting the attained numerical solutions with the analytical solutions. The author is aware of no studies that have been conducted on the suggested algorithm for TFDWE.

The outline of this research paper are: Parseval’s identity (PI) and CBS functions are included in section 2, and the proposed algorithm is discussed in section 3. The stability of current methodology and analysis of convergence are covered in sections 4 and 5, respectively. In section 6, the effectiveness and validity of the suggested technique are investigated, and finally, section 7 summarizes the conclusion.

## 2 Preliminaries

**Definition 1.** If $\tilde{g}\in L^{2}\left[a,b\right]$, then PI is defined as [60]:

$$\begin{array}{c} \sum_{m=-\infty }^{\infty }|\hat{g}{\left(m\right)|}^{2}=\int_{a}^{b}{\left|\tilde{g}\left(u\right)\right|}^{2}du, \end{array}$$ (5)

where $\hat{g}\left(m\right)=\int_{a}^{b}\tilde{g}\left(u\right)e^{2\pi imu}du$ is Fourier transform for all integers *m*.

### 2.1 Basis functions

Consider *a* = *u*_0_ < *u*_1_ < ⋯ < *u*_*N*_ = *b* be the equivalent partition of [*a*, *b*] at the knots *u*_*r*_ = *u*_0_ + *rh*, *r* = 0, 1, ⋯, *N*, where $h=\frac{b-a}{N}$. The CBS functions are expressed as [57]:

$$\begin{array}{c} S_{r}\left(u\right)=\frac{1}{6h^{3}}\{\begin{array}{cc} {\left(u-u_{r-2}\right)}^{3}, & \text{if}\;u\in [u_{r-2},u_{r-1}),\\ h^{3}+3h^{2}\left(u-u_{r-1}\right)+3h{\left(u-u_{r-1}\right)}^{2}-3{\left(u-u_{r-1}\right)}^{3}, & \text{if}\;u\in [u_{r-1},u_{r}),\\ h^{3}+3h^{2}\left(u_{r+1}-u\right)+3h{\left(u_{r+1}-u\right)}^{2}-3{\left(u_{r+1}-u\right)}^{3}, & \text{if}\;u\in [u_{r},u_{r+1}),\\ {\left(u_{r+2}-u\right)}^{3}, & \text{if}\;u\in [u_{r+1},u_{r+2}),\\ 0, & \text{otherwise}, \end{array} \end{array}$$ (6)

where *u* is the variable. The CBS has geometrical characteristics like non-negativity, convex hull property, symmetry, partition of unity and geometric invariability [53]. Additionally, *S*_−1_, *S*_0_, ⋯, *S*_*N*+1_ have been constructed. For *w*(*u*, *t*), the approximation *W*(*u*, *t*) in terms of CBS can be assumed as [59]:

$$\begin{array}{c} W\left(u,t\right)=\sum_{r=-1}^{N+1}\vartheta_{r}\left(t\right)S_{r}\left(u\right), \end{array}$$ (7)

where control points *ϑ*_*r*_(*t*) to be calculated at every temporal stage. Eqs (6) and (7) provide the following approximations at the nodal points:

$$\begin{array}{c} \left\{ \begin{array}{c} W\left(u_{r},t\right)={\left(W\right)}_{r}=(\frac{1}{6})\vartheta_{r-1}+(\frac{4}{6})\vartheta_{r}+(\frac{1}{6})\vartheta_{r+1},\\ W_{u}\left(u_{r},t\right)={\left(W_{u}\right)}_{r}=(-\frac{1}{2h})\vartheta_{r-1}+(\frac{1}{2h})\vartheta_{r+1},\\ W_{uu}\left(u_{r},t\right)={\left(W_{uu}\right)}_{r}=(\frac{1}{h^{2}})\vartheta_{r-1}+(-\frac{2}{h^{2}})\vartheta_{r}+(\frac{1}{h^{2}})\vartheta_{r+1}. \end{array}\right. \end{array}$$ (8)

## 3 Description of numerical scheme

Suppose the interval [0, *T*] be split into *M* equivalent sub-intervals of length $\Delta t=\frac{T}{M}$ utilizing the knots 0 = *t*_0_ < *t*_1_ < ⋯ < *t*_*M*_ = *T*, where *t*_*n*_ = *n*Δ*t*, *n* = 0, 1, ⋯, *M*. The CFFD of TFDWE is discretized at *t* = *t*_*n*+1_ as

$$\begin{array}{ccc} \frac{\partial^{\gamma }}{\partial t^{\gamma }}w\left(u,t_{n+1}\right) & = & \frac{R(\gamma )}{2-\gamma }\int_{0}^{t_{n+1}}\frac{\partial^{2}}{\partial v^{2}}w\left(u,v\right)\;\text{exp}\;[-\frac{\gamma }{2-\gamma }\left(t_{n+1}-v\right)]dv,\\ & = & \frac{R(\gamma )}{2-\gamma }\sum_{s=0}^{n}\int_{t_{s}}^{t_{s+1}}\frac{\partial^{2}}{\partial v^{2}}w\left(u,v\right)\;\text{exp}\;[-\frac{\gamma }{2-\gamma }\left(t_{n+1}-v\right)]dv.\;\;\;\;\;\; \end{array}$$ (9)

Using forward difference approach, Eq (9) becomes

$$\begin{array}{cc} \frac{\partial^{\gamma }}{\partial t^{\gamma }}w\left(u,t_{n+1}\right) & =\frac{R(\gamma )}{2-\gamma }\sum_{s=0}^{n}\frac{w\left(u,t_{s+1}\right)-2w\left(u,t_{s}\right)+w\left(u,t_{s-1}\right)}{{\left(\Delta t\right)}^{2}}\\ & \times \int_{t_{s}}^{t_{s+1}}\;\text{exp}\;[-\frac{\gamma }{2-\gamma }\left(t_{n+1}-v\right)]dv+\Phi_{\Delta t}^{n+1},\\ & =\frac{R(\gamma )}{\gamma {\left(\Delta t\right)}^{2}}[1-\;\text{exp}\;(-\frac{\gamma }{2-\gamma }\Delta t)]\sum_{s=0}^{n}[w\left(u,t_{n-s+1}\right)-2w\left(u,t_{n-s}\right)\\ & +w\left(u,t_{n-s-1}\right)]\;\text{exp}\;(-\frac{\gamma }{2-\gamma }s\Delta t)+\Phi_{\Delta t}^{n+1}. \end{array}$$

Hence

$$\begin{array}{c} \frac{\partial^{\gamma }}{\partial t^{\gamma }}w\left(u,t_{n+1}\right)=\frac{\mu R(\gamma )}{\gamma {\left(\Delta t\right)}^{2}}\sum_{s=0}^{n}k_{s}[w\left(u,t_{n-s+1}\right)-2w\left(u,t_{n-s}\right)+w\left(u,t_{n-s-1}\right)]+\Phi_{\Delta t}^{n+1}, \end{array}$$ (10)

where $\mu =1-\;\text{exp}\;(-\frac{\gamma }{2-\gamma }\Delta t)$ and $k_{s}=\;\text{exp}\;\left(-\frac{\gamma }{2-\gamma }s\Delta t\right)$. Moreover, the truncation error $\Phi_{\Delta t}^{n+1}$ is given as

$$\begin{array}{c} |\Phi_{\Delta t}^{n+1}{|\leq Ϝ\left(\Delta t\right)}^{2}, \end{array}$$ (11)

where Ϝ is a constant. It is straightforward to check that

- *k*_*s*_ > 0 and *k*_0_ = 1, *s* = 0, 1, ⋯, *n*,
- *k*_0_ > *k*_1_ > *k*_2_ > ⋯ > *k*_*s*_, *k*_*s*_ → 0 as *s* → ∞,
-
$\sum_{s=0}^{n}\left(k_{s}-k_{s+1}\right)+k_{n+1}=\left(1-k_{1}\right)+\sum_{s=1}^{n-1}\left(k_{s}-k_{s+1}\right)+k_{n}=1$ .

Using (10) and *θ*-weighted scheme, Eq (1) becomes

$$\begin{array}{c} \frac{\mu R(\gamma )}{\gamma {\left(\Delta t\right)}^{2}}\sum_{s=0}^{n}k_{s}[w\left(u,t_{n-s+1}\right)-2w\left(u,t_{n-s}\right)+w\left(u,t_{n-s-1}\right)]+\frac{\delta }{\Delta t}[w\left(u,t_{n+1}\right)-w\left(u,t_{n}\right)]\\ +\theta (\sigma w\left(u,t_{n+1}\right)-w_{uu}\left(u,t_{n+1}\right))+\left(1-\theta \right)(\sigma w\left(u,t_{n}\right)-w_{uu}\left(u,t_{n}\right))=q\left(u,t_{n+1}\right). \end{array}$$ (12)

Discretizing (12) for *θ* = 1, we achieve

$$\begin{array}{c} \left(\varrho +\delta_{0}+\sigma \right)w_{r}^{n+1}-\left(2\varrho +\delta_{0}\right)w_{r}^{n}+\varrho w_{r}^{n-1}+\varrho \sum_{s=1}^{n}k_{s}[w_{r}^{n-s+1}-2w_{r}^{n-s}+w_{r}^{n-s-1}]\\ -{\left(w_{uu}\right)}_{r}^{n+1}=q_{r}^{n+1},\;\;\;n=0,1,\cdots ,M, \end{array}$$ (13)

where $\varrho =\frac{\mu R(\gamma )}{\gamma {\left(\Delta t\right)}^{2}}$, $\delta_{0}=\frac{\delta }{\Delta t}$, $w_{r}^{n}=w\left(u_{r},t_{n}\right)$ and $q_{r}^{n+1}=q\left(u_{r},t_{n+1}\right)$. It is detected that the term *w*^−1^ will appear when *n* = *s* or *n* = 0. To remove *w*^−1^, we use the IC to attain

$$\begin{array}{c} w^{-1}=w^{1}-2\Delta t\psi_{2}\left(u\right). \end{array}$$ (14)

Employing the CBS approximation and its necessary derivatives at knot *u*_*r*_ in (13), then

$$\begin{array}{c} \left(\varrho +\delta_{0}+\sigma \right)W_{r}^{n+1}-{\left(W_{uu}\right)}_{r}^{n+1}\\ =\left(2\varrho +\delta_{0}\right)W_{r}^{n}-\varrho W_{r}^{n-1}-\varrho \sum_{s=1}^{n}k_{s}[W_{r}^{n-s+1}-2W_{r}^{n-s}+W_{r}^{n-s-1}]+q_{r}^{n+1}. \end{array}$$ (15)

Substituting (8) in (15), we get

$$\begin{array}{c} {}[\frac{1}{6}\left(\varrho +\delta_{0}+\sigma \right)-\frac{1}{h^{2}}]\vartheta_{r-1}^{n+1}+[\frac{4}{6}\left(\varrho +\delta_{0}+\sigma \right)+\frac{2}{h^{2}}]\vartheta_{r}^{n+1}+[\frac{1}{6}\left(\varrho +\delta_{0}+\sigma \right)-\frac{1}{h^{2}}]\vartheta_{r+1}^{n+1}\\ =\left(2\varrho +\delta_{0}\right)(\frac{1}{6}\vartheta_{r-1}^{n}+\frac{4}{6}\vartheta_{r}^{n}+\frac{1}{6}\vartheta_{r+1}^{n})-\varrho (\frac{1}{6}\vartheta_{r-1}^{n-1}+\frac{4}{6}\vartheta_{r}^{n-1}+\frac{1}{6}\vartheta_{r+1}^{n-1})\\ -\varrho \sum_{s=1}^{n}k_{s}[\frac{1}{6}\left(\vartheta_{r-1}^{n-s+1}-2\vartheta_{r-1}^{n-s}+\vartheta_{r-1}^{n-s-1}\right)+\frac{4}{6}\left(\vartheta_{r}^{n-s+1}-2\vartheta_{r}^{n-s}+\vartheta_{r}^{n-s-1}\right)\\ +\frac{1}{6}\left(\vartheta_{r+1}^{n-s+1}-2\vartheta_{r+1}^{n-s}+\vartheta_{r+1}^{n-s-1}\right)]+q_{r}^{n+1}. \end{array}$$ (16)

This system (16) has *N* + 1 linear equations involving *N* + 3 unknowns. To attain a consistent system, two additional equations are acquired by utilizing the BCs (3). Hence, a matrix system of dimension (*N* + 3) × (*N* + 3) is attained which can be uniquely solved utilizing any appropriate algorithm. Before using (16), the initial vector $\vartheta^{0}={\left[\vartheta_{-1}^{0},\vartheta_{0}^{0},\cdots ,\vartheta_{N+1}^{0}\right]}^{T}$ is achieved through the ICs as

$$\begin{array}{c} \left\{ \begin{array}{cc} {\left(W_{u}\right)}_{r}^{0}=\psi_{1}^{\prime }\left(u_{r}\right), & r=0,\\ {\left(W\right)}_{r}^{0}=\psi_{1}\left(u_{r}\right), & r=0,1,\cdots ,N,\\ {\left(W_{u}\right)}_{r}^{0}=\psi_{1}^{\prime }\left(u_{r}\right), & r=N. \end{array}\right. \end{array}$$ (17)

The matrix form of Eq (17) is expressed as

$$\begin{array}{c} K\vartheta^{0}=H, \end{array}$$ (18)

where

$$K=[\begin{array}{ccccccc} -\frac{1}{2h} & 0 & \frac{1}{2h} & & & & \\ \frac{1}{6} & \frac{4}{6} & \frac{1}{6} & & & & \\ & \frac{1}{6} & \frac{4}{6} & \frac{1}{6} & & & \\ & & \ddots & \ddots & \ddots & & \\ & & & \frac{1}{6} & \frac{4}{6} & \frac{1}{6} & \\ & & & & \frac{1}{6} & \frac{4}{6} & \frac{1}{6}\\ & & & & -\frac{1}{2h} & 0 & \frac{1}{2h} \end{array}]\;\text{and}\;H=[\begin{array}{c} \psi_{1}^{\prime }\left(u_{0}\right)\\ \psi_{1}\left(u_{0}\right)\\ \psi_{1}\left(u_{1}\right)\\ \vdots \\ \psi_{1}\left(u_{N-1}\right)\\ \psi_{1}\left(u_{N}\right)\\ \psi_{1}^{\prime }\left(u_{N}\right) \end{array}].$$

Any numerical algorithm can be used to solve Eq (18) for *ϑ*^0^. Mathematica 12 is utilized to accomplish the numerical results.

## 4 The stability of presented scheme

If the error does not increase while the computation is in progress, it can be inferred that the numerical method is stable [61]. Fourier method is employed for the stability of presented scheme. For this, suppose that $\varsigma_{r}^{n}$ and ${\tilde{\varsigma }}_{r}^{n}$ represent the growth factors analytically and numerically, respectively. The error $\varphi_{r}^{n}$ can be written as

$$\varphi_{r}^{n}=\varsigma_{r}^{n}-{\tilde{\varsigma }}_{r}^{n},\;\;\;\;r=1,\cdots ,N-1,\;\;pomcoln=0,1,\cdots ,M.$$

Thus from Eq (16), we obtain

$$\begin{array}{c} {}[\frac{1}{6}\left(\varrho +\delta_{0}+\sigma \right)-\frac{1}{h^{2}}]\varphi_{r-1}^{n+1}+[\frac{4}{6}\left(\varrho +\delta_{0}+\sigma \right)+\frac{2}{h^{2}}]\varphi_{r}^{n+1}+[\frac{1}{6}\left(\varrho +\delta_{0}+\sigma \right)-\frac{1}{h^{2}}]\varphi_{r+1}^{n+1}\\ =\left(2\varrho +\delta_{0}\right)(\frac{1}{6}\varphi_{r-1}^{n}+\frac{4}{6}\varphi_{r}^{n}+\frac{1}{6}\varphi_{r+1}^{n})-\varrho (\frac{1}{6}\varphi_{r-1}^{n-1}+\frac{4}{6}\varphi_{r}^{n-1}+\frac{1}{6}\varphi_{r+1}^{n-1})\\ -\varrho \sum_{s=1}^{n}k_{s}[\frac{1}{6}\left(\varphi_{r-1}^{n-s+1}-2\varphi_{r-1}^{n-s}+\varphi_{r-1}^{n-s-1}\right)+\frac{4}{6}\left(\varphi_{r}^{n-s+1}-2\varphi_{r}^{n-s}+\varphi_{r}^{n-s-1}\right)\\ +\frac{1}{6}\left(\varphi_{r+1}^{n-s+1}-2\varphi_{r+1}^{n-s}+\varphi_{r+1}^{n-s-1}\right)]. \end{array}$$ (19)

From ICs and BCs, we can write

$$\begin{array}{c} \varphi_{r}^{0}=\psi_{1}\left(u_{r}\right),\;\;\;{\left(\varphi_{t}\right)}_{r}^{0}=\psi_{2}\left(u_{r}\right),\;\;r=1,2,\cdots ,N \end{array}$$ (20)

and

$$\begin{array}{c} \varphi_{0}^{n}=\phi_{1}\left(t_{n}\right),\;\;\varphi_{N}^{n}=\varphi_{2}\left(t_{n}\right),\;\;n=0,1,\cdots ,M. \end{array}$$ (21)

The grid function is stated as:

$$\begin{array}{c} \varphi^{n}=\{\begin{array}{cc} \varphi_{r}^{n}, & u\in (u_{r}-\frac{h}{2},u_{r}+\frac{h}{2}],\;r=1,2,\cdots ,N-1,\\ 0, & u\in [a,a+\frac{h}{2}]\;\text{or}\;u\in [b-\frac{h}{2},b]. \end{array} \end{array}$$ (22)

The *φ*^*n*^(*u*) in the Fourier mode can be presented as:

$$\begin{array}{c} \varphi^{n}\left(u\right)=\sum_{m=-\infty }^{\infty }\chi^{n}\left(m\right)e^{\frac{2\pi imu}{b-a}}, \end{array}$$ (23)

where

$$\begin{array}{c} \chi^{n}\left(m\right)=\frac{1}{b-a}\int_{a}^{b}\varphi^{n}\left(u\right)e^{\frac{-2\pi imu}{b-a}}du,\;\;n=0\left(1\right)M\;\;\text{and}\;\;\varphi^{n}={\left[\varphi_{1}^{n},\varphi_{2}^{n},\cdots ,\varphi_{N-1}^{n}\right]}^{T}. \end{array}$$ (24)

Implementing ‖.‖_2_ norm, we attain

$$\begin{array}{cc} \|\varphi^{n}{\|}_{2} & =\sqrt{\sum_{r=1}^{N-1}h{\left|\varphi_{r}^{n}\right|}^{2}},\\ & ={\left(\int_{a}^{a+\frac{h}{2}}|\varphi^{n}{|}^{2}du+\sum_{r=1}^{N-1}\int_{u_{r}-\frac{h}{2}}^{u_{r}+\frac{h}{2}}|\varphi^{n}{|}^{2}du+\int_{b-\frac{h}{2}}^{b}{\left|\varphi^{n}\right|}^{2}du\right)}^{\frac{1}{2}},\\ & ={\left(\int_{a}^{b}{\left|\varphi^{n}\right|}^{2}du\right)}^{\frac{1}{2}}. \end{array}$$

Using Parseval’s identity (5), we achieve

$$\int_{a}^{b}|\varphi^{n}{|}^{2}du=\sum_{m=-\infty }^{\infty }{\left|\chi^{n}\left(m\right)\right|}^{2}.$$

Hence, we acquire

$$\begin{array}{c} \|\varphi^{n}{\|}_{2}^{2}=\sum_{m=-\infty }^{\infty }{\left|\chi^{n}\left(m\right)\right|}^{2}. \end{array}$$ (25)

Assume that the Eqs (19)–(21) possess the solution in Fourier sense as:

$$\begin{array}{c} \varphi_{r}^{n}=\chi^{n}e^{i\rho rh}, \end{array}$$ (26)

where $i=\sqrt{-1}$ and *ρ* is any real number. Substituting (26) in (19) and simplifying, we achieve

$$\begin{array}{c} {}[\frac{1}{6}\left(\varrho +\delta_{0}+\sigma \right)-\frac{1}{h^{2}}]\chi^{n+1}e^{-i\rho h}+[\frac{4}{6}\left(\varrho +\delta_{0}+\sigma \right)+\frac{2}{h^{2}}]\chi^{n+1}+[\frac{1}{6}\left(\varrho +\delta_{0}+\sigma \right)\\ -\frac{1}{h^{2}}]\chi^{n+1}e^{i\rho h}\\ =\left(2\varrho +\delta_{0}\right)(\frac{1}{6}\chi^{n}e^{-i\rho h}+\frac{4}{6}\chi^{n}+\frac{1}{6}\chi^{n}e^{i\rho h})-(\frac{\varrho }{6}\chi^{n-1}e^{-i\rho h}+\frac{4\varrho }{6}\chi^{n-1}+\frac{\varrho }{6}\chi^{n-1}e^{i\rho h})\\ -\sum_{s=1}^{n}k_{s}[\frac{\varrho }{6}(\chi^{n-s+1}e^{-i\rho h}-2\chi^{n-s}e^{-i\rho h}+\chi^{n-s-1}e^{-i\rho h})+\frac{4\varrho }{6}(\chi^{n-s+1}-2\chi^{n-s}\\ +\chi^{n-s-1})+\frac{\varrho }{6}(\chi^{n-s+1}e^{i\rho h}-2\chi^{n-s}e^{i\rho h}+\chi^{n-s-1}e^{i\rho h})]. \end{array}$$ (27)

Utilizing the relation *e*^*iρh*^ + *e*^−*iρh*^ = 2*cos*(*ρh*) and simplifying, we get

$$\begin{array}{c} \chi^{n+1}=\frac{\left(1+ℵ\right)}{\varepsilon }\chi^{n}-\frac{ℵ}{\varepsilon }\chi^{n-1}-\frac{ℵ}{\varepsilon }\sum_{s=1}^{n}k_{s}(\chi^{n-s+1}-2\chi^{n-s}+\chi^{n-s-1}), \end{array}$$ (28)

where $ℵ=\frac{\varrho }{\varrho +\delta_{0}}$ and $\varepsilon =1+\frac{2\;\text{cos}\;\left(\rho h\right)\left(\sigma h^{2}-6\right)+4\sigma h^{2}+12}{\left(\varrho h^{2}+\delta_{0}h^{2}\right)\left(2\;\text{cos}\;\left(\rho h\right)+4\right)}$. Obviously *ε* ≥ 1.

**Proposition 4.1**. *If* *χ*^*n*^ *is the solution for* Eq (28), *then* |*χ*^*n*^| ≤ (1 + ℵ)|*χ*^0^|, *n* = 0, 1, ⋯, *M*.

*Proof.* To prove this, mathematical induction is utilized. For *n* = 0, Eq (28) gives

$$|\chi^{1}|=\frac{\left(1+ℵ\right)}{\varepsilon }|\chi^{0}\left|\leq \left(1+ℵ\right)\right|\chi^{0}|,\;\;\;\;\varepsilon \geq 1.$$

Assume that |*χ*^*n*^| ≤ (1 + ℵ)|*χ*^0^| for *n* = 1, 2, ⋯, *M* − 1, then

$$\begin{array}{cc} |\chi^{n+1}| & \leq \frac{\left(1+ℵ\right)}{\varepsilon }|\chi^{n}|-\frac{ℵ}{\varepsilon }\left|\chi^{n-1}\right|-\frac{ℵ}{\varepsilon }\sum_{s=1}^{n}k_{s}(|\chi^{n-s+1}\left|-2\right|\chi^{n-s}\left|+\right|\chi^{n-s-1}|),\\ & \leq \frac{{\left(1+ℵ\right)}^{2}}{\varepsilon }|\chi^{0}|-\frac{ℵ(1+ℵ)}{\varepsilon }\left|\chi^{0}\right|-\frac{ℵ(1+ℵ)}{\varepsilon }\sum_{s=1}^{n}k_{s}(|\chi^{0}\left|-2\right|\chi^{0}\left|+\right|\chi^{0}|),\\ & =\frac{\left(1+ℵ\right)}{\varepsilon }\left[1+ℵ-ℵ\right]\left|\chi^{0}\right|,\\ |\chi^{n+1}| & \leq \left(1+ℵ\right)|\chi^{0}|. \end{array}$$

**Theorem 1**. *The proposed method* (16) *is unconditionally stable*.

*Proof.* Employing Eq (25) and Proposition 4.1, we get

$$\|\varphi^{n}{\|}_{2}\leq \left(1+ℵ\right){\left\|\varphi^{0}\right\|}_{2},\;\;\;n=0,1,\cdots ,M.$$

Thus, the proposed computational technique is stable unconditionally.

## 5 The convergence of presented scheme

The methodology given in [62] is used to investigate the convergence of the suggested approach. First and foremost, the following theorem is presented as [63, 64]:

**Theorem 2**. *Suppose* *q* *belongs to* *C*^2^[*a*, *b*] *and* *w*(*u*, *t*) *belongs to* *C*^4^[*a*, *b*], *also partition of* [*a*, *b*] *is* *Υ* = {*a* = *u*_0_, *u*_1_, ⋯, *u*_*N*_ = *b*} *with* *u*_*r*_ = *a* + *rh*, where *r* = 0, 1, ⋯, *N*. *If solution curve is interpolated by unique spline* $\tilde{W}\left(u,t\right)$ *at* *u*_*r*_ ∈ ϒ, *then for every* *t* ≥ 0 *there exist* *ξ*_*r*_ *independent of* *h*, *for* *r* = 0, 1, 2, *we have*

$$\begin{array}{c} \|D^{r}(w\left(u,t\right)-\tilde{W}\left(u,t\right)){\|}_{\infty }\leq \xi_{r}h^{4-r}. \end{array}$$ (29)

**Lemma 5.1.** *The CBS set* {*S*_−1_, *S*_0_, ⋯, *S*_*N* + 1_} *in* (6) *fulfils the inequality as given in* [21]

$$\begin{array}{c} \sum_{r=-1}^{N+1}\left|S_{r}\left(u\right)\right|\leq \frac{5}{3},\;\;\;0\leq u\leq 1. \end{array}$$ (30)

**Theorem 3**. *The numerical solution* *W*(*u*, *t*) *to the exact solution* *w*(*u*, *t*) *for TFDWE* (1)–(3) *exists. Furthermore, if* *q* *is a member of* *C*^2^[0, 1], *then*

$$\begin{array}{c} {\left\|w\left(u,t\right)-W\left(u,t\right)\right\|}_{\infty }\leq \tilde{\xi }h^{2},\;\;\;\forall \;t\geq 0, \end{array}$$ (31)

*where* *h* *is suitably small and* $\tilde{\xi }>0$ *is independent of* *h*.

*Proof.* Let $\tilde{W}\left(u,t\right)=\sum_{r=-1}^{N+1}x_{r}^{n}\left(t\right)S_{r}\left(u\right)$ be the estimated CBS for *W*(*u*, *t*). Through the triangular inequality, we get

$${\left\|w\left(u,t\right)-W\left(u,t\right)\right\|}_{\infty }\leq \|w\left(u,t\right)-\tilde{W}{\left(u,t\right)\|}_{\infty }+{\left\|\tilde{W}\left(u,t\right)-W\left(u,t\right)\right\|}_{\infty }.$$

With the aid of Theorem 2 for *r* = 0, we attain

$$\begin{array}{c} {\left\|w\left(u,t\right)-W\left(u,t\right)\right\|}_{\infty }\leq \xi_{0}h^{4}+{\left\|\tilde{W}\left(u,t\right)-W\left(u,t\right)\right\|}_{\infty }. \end{array}$$ (32)

The present method possesses collocation conditions *Lw*(*u*_*r*_, *t*) = *LW*(*u*_*r*_, *t*) = *q*(*u*_*r*_, *t*), *r* = 0, 1, ⋯, *N*. Consider

$$L\tilde{W}\left(u_{r},t\right)=\tilde{q}\left(u_{r},t\right).$$

Therefore, the difference equation $L(\tilde{W}\left(u_{r},t\right)-W\left(u_{r},t\right))$ can be stated at time level n as

$$\begin{array}{c} {}[\frac{1}{6}\left(\varrho +\delta_{0}+\sigma \right)-\frac{1}{h^{2}}]\Omega_{r-1}^{n+1}+[\frac{4}{6}\left(\varrho +\delta_{0}+\sigma \right)+\frac{2}{h^{2}}]\Omega_{r}^{n+1}+[\frac{1}{6}\left(\varrho +\delta_{0}+\sigma \right)-\frac{1}{h^{2}}]\Omega_{r+1}^{n+1}\\ =\left(2\varrho +\delta_{0}\right)(\frac{1}{6}\Omega_{r-1}^{n}+\frac{4}{6}\Omega_{r}^{n}+\frac{1}{6}\Omega_{r+1}^{n})-\varrho (\frac{1}{6}\Omega_{r-1}^{n-1}+\frac{4}{6}\Omega_{r}^{n-1}+\frac{1}{6}\Omega_{r+1}^{n-1})\\ -\varrho \sum_{s=1}^{n}k_{s}[\frac{1}{6}\left(\Omega_{r-1}^{n-s+1}-2\Omega_{r-1}^{n-s}+\Omega_{r-1}^{n-s-1}\right)+\frac{4}{6}\left(\Omega_{r}^{n-s+1}-2\Omega_{r}^{n-s}+\Omega_{r}^{n-s-1}\right)\\ +\frac{1}{6}\left(\Omega_{r+1}^{n-s+1}-2\Omega_{r+1}^{n-s}+\Omega_{r+1}^{n-s-1}\right)]+\frac{1}{h^{2}}\zeta_{r}^{n+1}. \end{array}$$ (33)

The BCs can be given as

$$\frac{1}{6}\Omega_{r-1}^{n+1}+\frac{4}{6}\Omega_{r}^{n+1}+\frac{1}{6}\Omega_{r+1}^{n+1}=0,\;\;\;r=0,N,$$

where

$$\Omega_{r}^{n}=\vartheta_{r}^{n}-x_{r}^{n},\;\;\;r=-1,0,\cdots ,N+1$$

and

$$\zeta_{r}^{n}=h^{2}\left[q_{r}^{n}-{\tilde{q}}_{r}^{n}\right],\;\;\;r=0,1,\cdots ,N.$$

From inequality (29), we achieve

$$|\zeta_{r}^{n}|=h^{2}\left|q_{r}^{n}-{\tilde{q}}_{r}^{n}\right|\leq \xi h^{4}.$$

Define $\zeta^{n}=\max_{0\leq r\leq N}\left|\zeta_{r}^{n}\right|$, $e_{r}^{n}=\left|\Omega_{r}^{n}\right|$ and $e^{n}=\max_{0\leq r\leq N}\left|e_{r}^{n}\right|$. When *n* = 0 and using the expression (14), Eq (33) becomes

$$\begin{array}{c} {}[\frac{1}{6}\left(\delta_{0}+\sigma \right)-\frac{1}{h^{2}}]\Omega_{r-1}^{1}+[\frac{4}{6}\left(\delta_{0}+\sigma \right)+\frac{2}{h^{2}}]\Omega_{r}^{1}+[\frac{1}{6}\left(\delta_{0}+\sigma \right)-\frac{1}{h^{2}}]\Omega_{r+1}^{1}\\ =\delta_{0}(\frac{1}{6}\Omega_{r-1}^{0}+\frac{4}{6}\Omega_{r}^{0}+\frac{1}{6}\Omega_{r+1}^{0})+\frac{1}{h^{2}}\zeta_{r}^{1}, \end{array}$$

where *r* = 0, 1, ⋯, *N*. Using IC, *e*^0^ = 0:

$$[\frac{4}{6}\left(\delta_{0}+\sigma \right)+\frac{2}{h^{2}}]\Omega_{r}^{1}=-[\frac{1}{6}\left(\delta_{0}+\sigma \right)-\frac{1}{h^{2}}]\left(\Omega_{r-1}^{1}+\Omega_{r+1}^{1}\right)+\frac{1}{h^{2}}\zeta_{r}^{1}.$$

Taking absolute values of $\Omega_{r}^{1}$, $\zeta_{r}^{1}$ and appropriately small *h*, we have

$$e_{r}^{1}\leq \frac{3\xi h^{4}}{h^{2}\left(\delta_{0}+\sigma \right)+12},\;\;\;0\leq r\leq N.$$

We get the values of $e_{-1}^{1}$ and $e_{N+1}^{1}$ through BCs:

$$e_{-1}^{1}\leq \frac{15\xi h^{4}}{h^{2}\left(\delta_{0}+\sigma \right)+12},$$

$$e_{N+1}^{1}\leq \frac{15\xi h^{4}}{h^{2}\left(\delta_{0}+\sigma \right)+12},$$

which implies

$$e^{1}\leq \xi_{1}h^{2},$$

where *ξ*_1_ is not depending on *h*. Now, mathematical induction is utilized for the proof of this theorem. Suppose that $e_{r}^{y}\leq \xi_{y}h^{2}$ is true for 1 ≤ *y* ≤ *n* and *ξ* = *max*{*ξ*_*y*_ : *y* = 0, 1, ⋯, *n*}, then from Eq (33), we acquire

$$\begin{array}{c} {}[\frac{1}{6}\left(\varrho +\delta_{0}+\sigma \right)-\frac{1}{h^{2}}]\Omega_{r-1}^{n+1}+[\frac{4}{6}\left(\varrho +\delta_{0}+\sigma \right)+\frac{2}{h^{2}}]\Omega_{r}^{n+1}+[\frac{1}{6}\left(\varrho +\delta_{0}+\sigma \right)-\frac{1}{h^{2}}]\Omega_{r+1}^{n+1}\\ =\left(2\varrho +\delta_{0}-\varrho k_{1}\right)(\frac{1}{6}\Omega_{r-1}^{n}+\frac{4}{6}\Omega_{r}^{n}+\frac{1}{6}\Omega_{r+1}^{n})-\varrho [\left(k_{0}-2k_{1}+k_{2}\right)(\frac{1}{6}\Omega_{r-1}^{n-1}+\frac{4}{6}\Omega_{r}^{n-1}\\ +\frac{1}{6}\Omega_{r+1}^{n-1})+\left(k_{1}-2k_{2}+k_{3}\right)(\frac{1}{6}\Omega_{r-1}^{n-2}+\frac{4}{6}\Omega_{r}^{n-2}+\frac{1}{6}\Omega_{r+1}^{n-2})+\left(k_{2}-2k_{3}+k_{4}\right)(\frac{1}{6}\Omega_{r-1}^{n-3}\\ +\frac{4}{6}\Omega_{r}^{n-3}+\frac{1}{6}\Omega_{r+1}^{n-3})+\cdots +\left(k_{n-3}-2k_{n-2}+k_{n-1}\right)(\frac{1}{6}\Omega_{r-1}^{2}+\frac{4}{6}\Omega_{r}^{2}+\frac{1}{6}\Omega_{r+1}^{2})\\ +\left(k_{n-2}-2k_{n-1}+k_{n}\right)(\frac{1}{6}\Omega_{r-1}^{1}+\frac{4}{6}\Omega_{r}^{1}+\frac{1}{6}\Omega_{r+1}^{1})+k_{n-1}(\frac{1}{6}\Omega_{r-1}^{0}+\frac{4}{6}\Omega_{r}^{0}+\frac{1}{6}\Omega_{r+1}^{0})\\ -k_{n}(\frac{1}{6}\Omega_{r-1}^{1}+\frac{4}{6}\Omega_{r}^{1}+\frac{1}{6}\Omega_{r+1}^{1})]+\frac{1}{h^{2}}\zeta_{r}^{n+1}. \end{array}$$

Again, taking absolute values of $\Omega_{r}^{n+1}$ and $\zeta_{r}^{n+1}$, we obtain

$$e_{r}^{n+1}\leq \frac{3h^{2}}{h^{2}\left(\varrho +\delta_{0}+\sigma \right)+12}[\left(2\varrho +\delta_{0}-\varrho k_{1}\right)\xi h^{2}-\varrho \sum_{s=1}^{n-1}\left(k_{s-1}-2k_{s}+k_{s+1}\right)\xi h^{2}+\varrho k_{n}\xi h^{2}+\xi h^{2}].$$

Similarly, we get the values of $e_{-1}^{n+1}$ and $e_{N+1}^{n+1}$ from the boundary conditions

$$\begin{array}{c} e_{-1}^{n+1}\leq \frac{15h^{2}}{h^{2}\left(\varrho +\delta_{0}+\sigma \right)+12}[\left(2\varrho +\delta_{0}-\varrho k_{1}\right)\xi h^{2}-\varrho \sum_{s=1}^{n-1}\left(k_{s-1}-2k_{s}+k_{s+1}\right)\xi h^{2}\\ +\varrho k_{n}\xi h^{2}+\xi h^{2}] \end{array}$$

and

$$\begin{array}{c} e_{N+1}^{n+1}\leq \frac{15h^{2}}{h^{2}\left(\varrho +\delta_{0}+\sigma \right)+12}[\left(2\varrho +\delta_{0}-\varrho k_{1}\right)\xi h^{2}-\varrho \sum_{s=1}^{n-1}\left(k_{s-1}-2k_{s}+k_{s+1}\right)\xi h^{2}\\ +\varrho k_{n}\xi h^{2}+\xi h^{2}]. \end{array}$$

Hence, for all *n*, we acquire

$$\begin{array}{c} e^{n+1}\leq \xi h^{2}. \end{array}$$ (34)

In particular,

$$\tilde{W}\left(u,t\right)-W\left(u,t\right)=\sum_{r=-1}^{N+1}(x_{r}\left(t\right)-\vartheta_{r}\left(t\right))S_{r}\left(u\right).$$

Therefore, from Lemma 5.1 and inequality (34), we get

$$\begin{array}{c} \|\tilde{W}{\left(u,t\right)-W\left(u,t\right)\|}_{\infty }\leq \frac{5}{3}\xi h^{2}. \end{array}$$ (35)

Using (35), the inequality (32) becomes

$${\left\|w\left(u,t\right)-W\left(u,t\right)\right\|}_{\infty }\leq \xi_{0}h^{4}+\frac{5}{3}\xi h^{2}=\tilde{\xi }h^{2},$$

where $\tilde{\xi }=\xi_{0}h^{2}+\frac{5}{3}\xi$.

**Theorem 4**. *The TFDWE is convergent with ICs and BCs*.

*Proof*. Assume that *w*(*u*, *t*) and *W*(*u*, *t*) are analytical and approximate results for TFDWE, respectively. Therefore, the preceding theorem and relation (11) justify that there exist constants $\tilde{\xi }$ and Ϝ such that

$${\left\|w\left(u,t\right)-W\left(u,t\right)\right\|}_{\infty }\leq \tilde{\xi }h^{2}+Ϝ{\left(\Delta t\right)}^{2}.$$

Thus, the proposed method is second order convergent.

## 6 Numerical results and discussion

In this section, numerical results of some experiments using proposed approach are demonstrated. To examine the validity of the proposed scheme, we employ error norms *L*_2_ and *L*_∞_ as

$$L_{\infty }=\|w\left(u_{r},t\right)-W\left(u_{r},t\right){\|}_{\infty }=\underset{0\leq r\leq N}{\text{max}}\;\left|w\left(u_{r},t\right)-W\left(u_{r},t\right)\right|$$

and

$$L_{2}={\left\|w\left(u_{r},t\right)-W\left(u_{r},t\right)\right\|}_{2}=\sqrt{h\sum_{r=0}^{N}{\left|w\left(u_{r},t\right)-W\left(u_{r},t\right)\right|}^{2}}.$$

Moreover, the convergence order is computed as

$${\text{log}}_{2}(\frac{L_{\infty }\left(N\right)}{L_{\infty }\left(2N\right)}).$$

All examples are examined by taking *R*(*γ*) = 1. Numerical calculations are carried out by utilizing Mathematica 12 on an Intel(R) Core(TM) i5-3437U CPU @ 1.90GHz 2.40 GHz with 12.0 GB RAM, SSD and 64-bit operating system (Windows 10).

**Example 6.1**. *Consider the TFDWE*

$$\frac{\partial^{\gamma }w\left(u,t\right)}{\partial t^{\gamma }}-\frac{\partial^{2}w\left(u,t\right)}{\partial u^{2}}=q\left(u,t\right),\;\;\gamma \in \left(1,2\right),\;\;u\in \left[0,1\right],\;\;t\in \left[0,1\right],$$

*with ICs*

$$w\left(u,0\right)=0,\;\;\;\;w_{t}\left(u,0\right)=-\text{sin}\left(\pi u\right)$$

*and the BCs*

w(0,t)=0,w(1,t)=0,

*where* $q\left(u,t\right)=2(\frac{R(\gamma )}{\gamma })\text{sin}\left(\pi u\right)(1-\;\text{exp}\;[-\frac{\gamma }{2-\gamma }t])+\pi^{2}\left(t^{2}-t\right)\text{sin}\left(\pi u\right)$.

**Fig 1:**
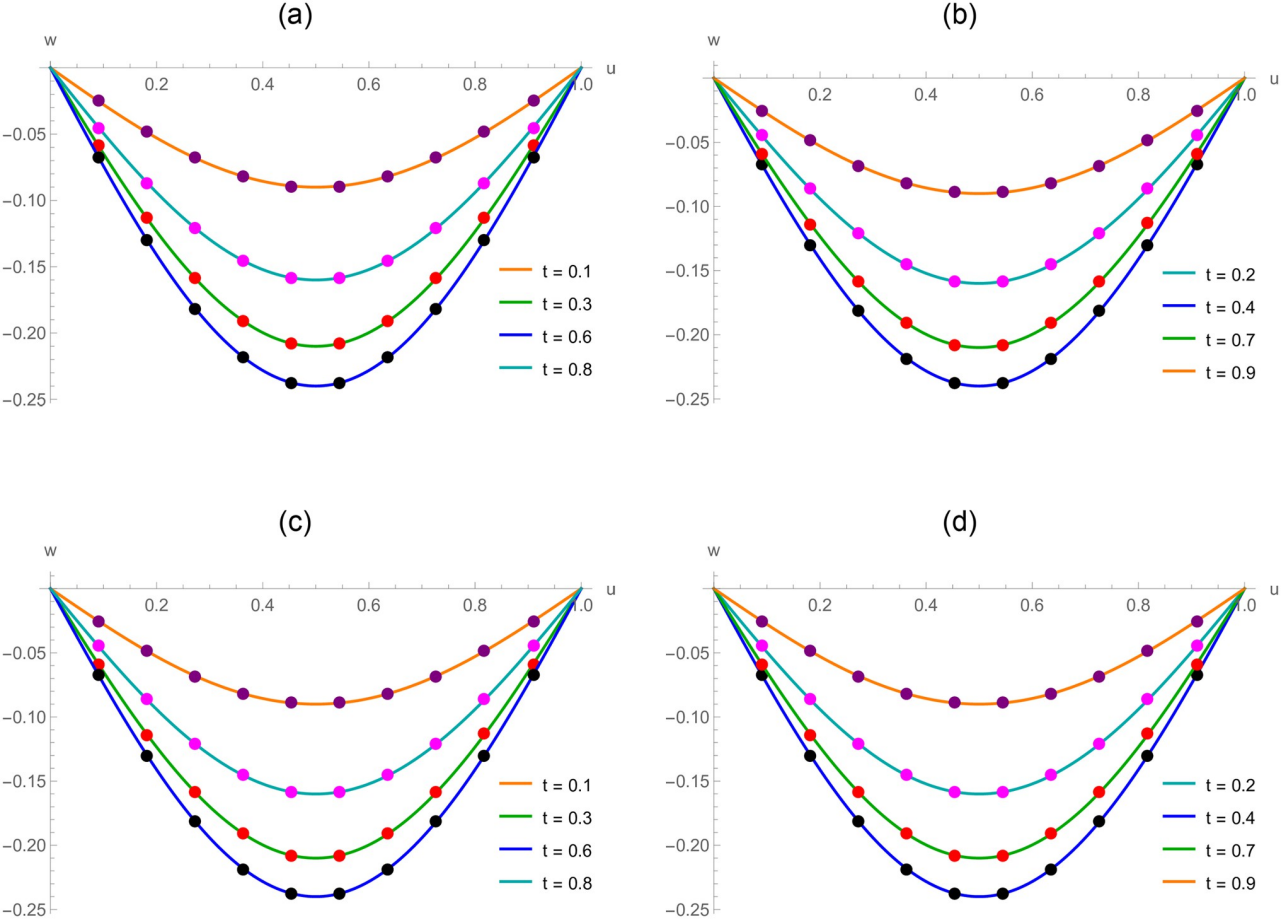
For Example 6.1, approximate and exact results at various temporal directions. (a) *N* = 250, *γ* = 1.8 and Δ*t* = 0.01 (b) *N* = 250, *γ* = 1.8 and Δ*t* = 0.01 (c) *N* = 165, *γ* = 1.5 and Δ*t* = 0.005 (d) *N* = 165, *γ* = 1.5 and Δ*t* = 0.005.

**Fig 2:**
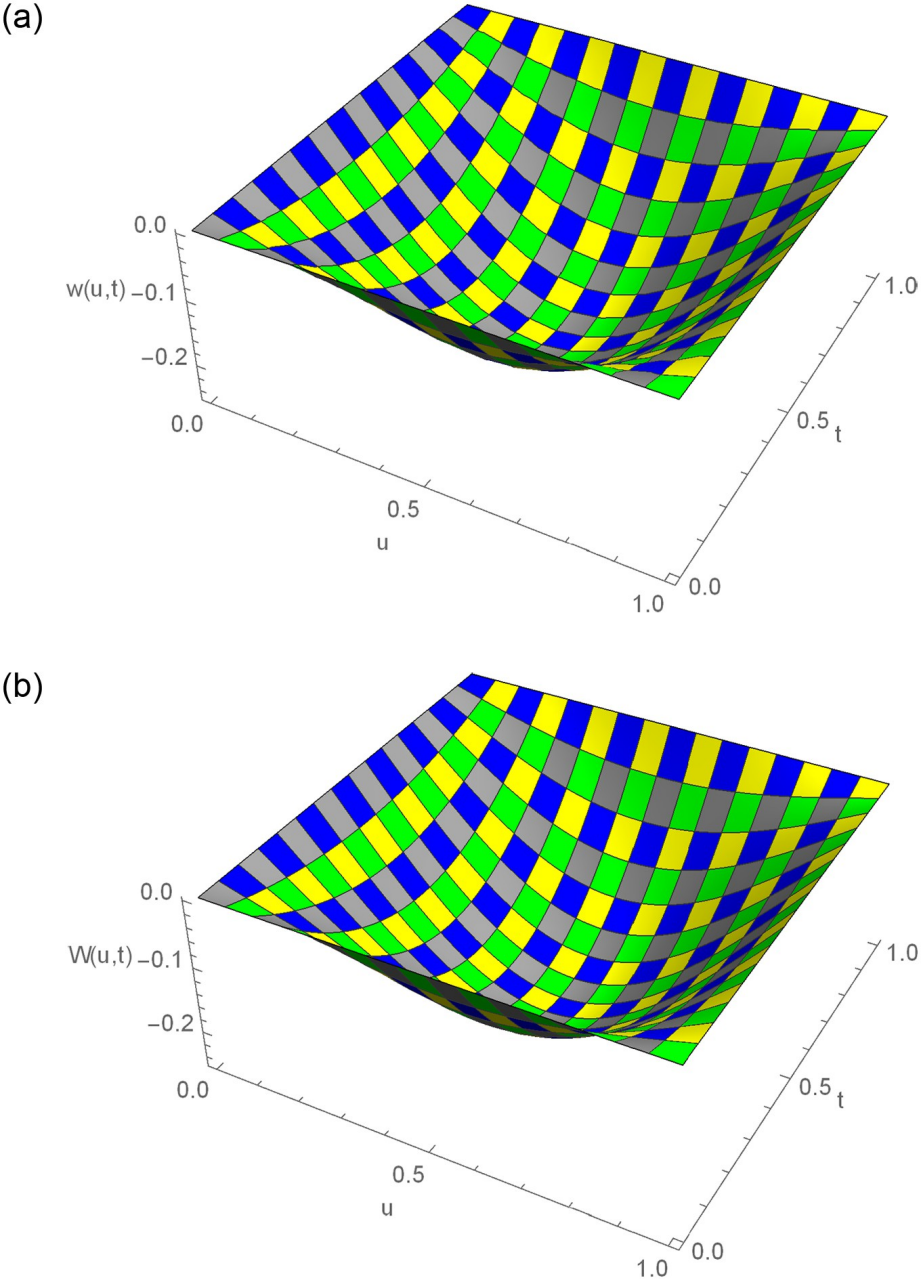
3D exact and numerical solutions for Example 6.1, where *t* = 1, *N* = 180, Δ*t* = 0.01, *γ* = 1.5 and *u* ∈ [0, 1]. (a) Exact result (b) Approximate result.

**Fig 3:**
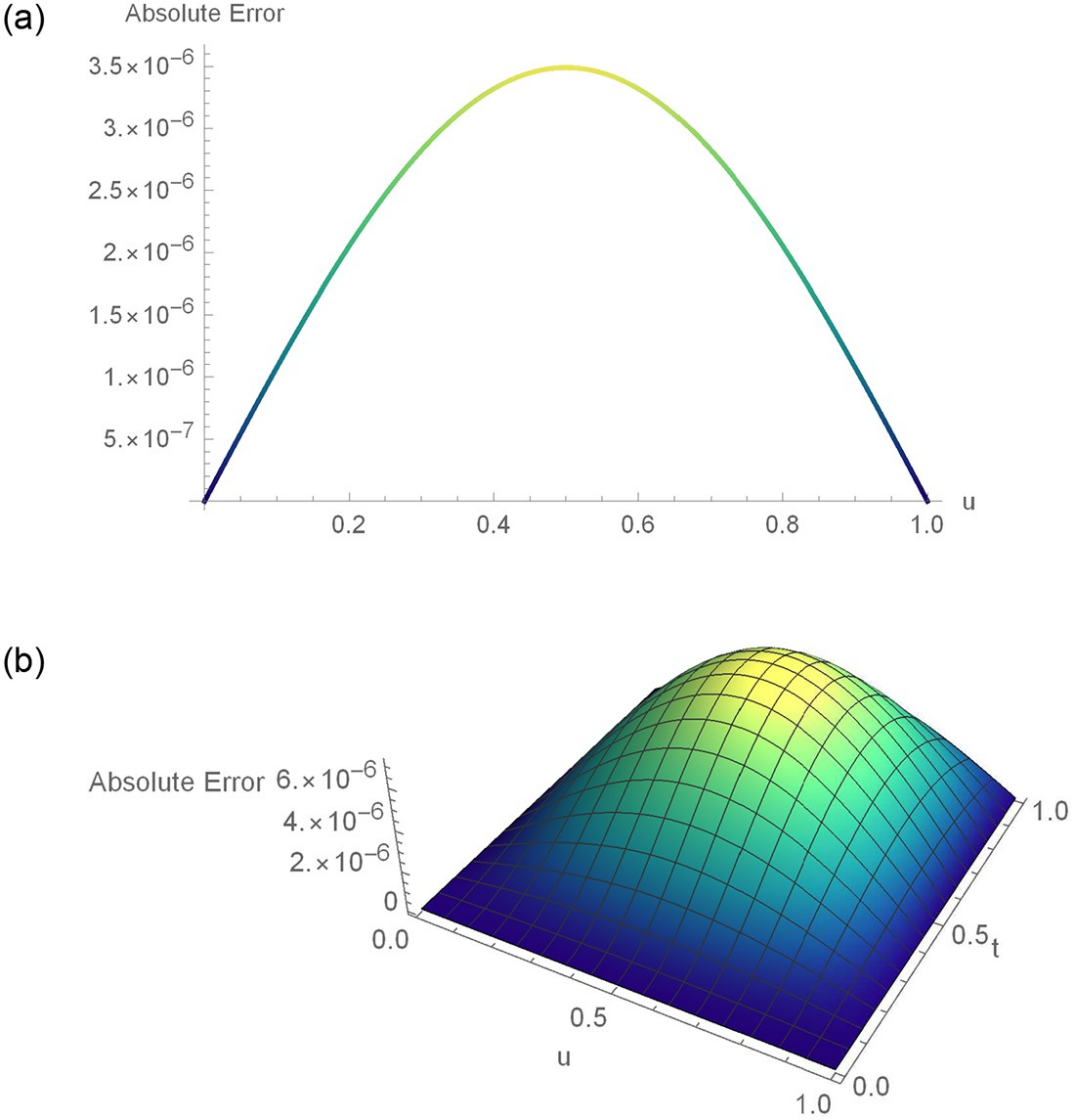
2D and 3D error profiles for Δ*t* = 0.01, *N* = 180,*γ* = 1.5, *u* ∈ [0, 1] and *t* = 1 for Example 6.1. (a) 2D error function (b) 3D error function.

The *w*(*u*, *t*) = (*t*^2^ − *t*)sin(*πu*) is the exact solution. The approximate results and absolute error for distinct *u* values at *t* = 0.2 for Example 6.1 are demonstrated in Table 1. For various time stages, error norms *L*_2_ and *L*_∞_ are displayed in Table 2. Table 3 contains the analysis of error norms and convergence order. Tables 4 and 5 show the error norms at numerous values of Δ*t* and *h*. The relation between numerical outcomes and analytical solutions at various levels of time is expressed in Fig 1. A 3D plot of numerical and analytical solutions is appeared in Fig 2. The graphs of errors in 2D and 3D forms are shown in Fig 3. Tables and graphics demonstrate that the propounded methodology gives a strong correlation with existing exact solution. The piecewise CBS numerical solutions when *γ* = 1.6, *N* = 50, Δ*t* = 0.001, *t* = 0.3 and *γ* = 1.9, *N* = 20, Δ*t* = 0.002 and *t* = 0.9 of Example 6.1, are given in Eqs (36) and (37), respectively.

$$\begin{array}{c} W\left(u,0.3\right)=\{\begin{array}{cc} -3.28257\times 10^{-14}+u(-0.659602+u(-6.79279\times 10^{-11}+1.08464u)), & \text{if}\;u\in [0.00,0.02)\\ 3.42447\times 10^{-8}+u(-0.659607+u(0.000256836+1.08036u)), & \text{if}\;u\in [0.02,0.04)\\ 5.81080\times 10^{-7}+u(-0.659648+u(0.00128215+1.07182u)), & \text{if}\;u\in [0.04,0.06)\\ 3.34032\times 10^{-6}+u(-0.659786+u(0.00358152+1.05905u)), & \text{if}\;u\in [0.06,0.08)\\ 1.20207\times 10^{-5}+u(-0.660111+u(0.00765045+1.04209u)), & \text{if}\;u\in [0.08,0.10)\\ 3.30872\times 10^{-5}+u(-0.660743+u(0.0139704+1.02102u)), & \text{if}\;u\in [0.10,0.12)\\ 7.64531\times 10^{-5}+u(-0.661828+(0.023005+0.995929u)u), & \text{if}\;u\in [0.12,0.14)\\ 0.000156102+u(-0.663534+(0.0351961+0.966902u)u), & \text{if}\;u\in [0.14,0.16)\\ \;\vdots \\ 0.0307839+u(-0.920914+(0.79400+0.16976u)u), & \text{if}\;u\in [0.44,0.46)\\ 0.0373672+u(-0.963848+(0.887337+0.102124u)u), & \text{if}\;u\in [0.46,0.48)\\ 0.0448917+u(-1.01088+(0.985311+0.0340863u)u), & \text{if}\;u\in [0.48,0.50)\\ 0.0534133+u(-1.06201+(1.08757-0.0340863u)u), & \text{if}\;u\in [0.50,0.52)\\ 0.0629800+u(-1.11720+(1.19371-0.102124u)u), & \text{if}\;u\in [0.52,0.54)\\ \;\vdots \\ 0.33872+u(-2.30757+(2.93590-0.966902u)u), & \text{if}\;u\in [0.84,0.86)\\ 0.357183+u(-2.37197+(3.01079-0.995929u)u), & \text{if}\;u\in [0.86,0.88)\\ 0.374285+u(-2.43027+(3.07705-1.02102u)u), & \text{if}\;u\in [0.88,0.90)\\ 0.389643+u(-2.48146+(3.13392-1.04209u)u), & \text{if}\;u\in [0.90,0.92)\\ 0.402844+u(-2.52451+(3.18072-1.05905u)u), & \text{if}\;u\in [0.92,0.94)\\ 0.413454+u(-2.55838+(3.21674-1.07182u)u), & \text{if}\;u\in [0.94,0.96)\\ 0.421014+u(-2.58200+(3.24135-1.08036u)u), & \text{if}\;u\in [0.96,0.98)\\ 0.425043+u(-2.59433+(3.25393-1.08464u)u), & \text{if}\;u\in [0.98,1.00). \end{array} \end{array}$$ (36)

and

$$\begin{array}{c} W\left(u,0.9\right)=\{\begin{array}{cc} -3.02319\times 10^{-15}+u(-0.280480+(-5.46496\times 10^{-12}+0.460423u)u), & \text{if}\;u\in [0.00,0.05)\\ 1.41714\times 10^{-6}+u(-0.280565+(0.00170057+0.449085u)u), & \text{if}\;u\in [0.05,0.10)\\ 2.38122\times 10^{-5}+u(-0.281236+(0.0084191+0.426690u)u), & \text{if}\;u\in [0.10,0.15)\\ 0.000134855+u(-0.283457+(0.0232248+0.393789u)u), & \text{if}\;u\in [0.15,0.20)\\ 0.000475639+u(-0.288569+(0.0487836+0.351191u)u), & \text{if}\;u\in [0.20,0.25)\\ 0.00127635+u(-0.298178+(0.0872178+0.299945u)u), & \text{if}\;u\in [0.25,0.30)\\ 0.00285939+u(-0.314008+(0.139986+0.241314u)u), & \text{if}\;u\in [0.30,0.35)\\ 0.00562796+u(-0.337739+(0.207788+0.176741u)u), & \text{if}\;u\in [0.35,0.40)\\ 0.0100392+u(-0.370823+(0.290498+0.107816u)u), & \text{if}\;u\in [0.40,0.45)\\ 0.0165619+u(-0.414307+(0.38713+0.036236u)u), & \text{if}\;u\in [0.45,0.50)\\ 0.0256209+u(-0.468661+(0.495838-0.036236u)u), & \text{if}\;u\in [0.50,0.55)\\ 0.0375300+u(-0.533620+(0.613945-0.107816u)u), & \text{if}\;u\in [0.55,0.60)\\ 0.0524178+u(-0.608059+(0.73801-0.176741u)u), & \text{if}\;u\in [0.60,0.65)\\ 0.0701512+u(-0.689906+(0.863928-0.241314u)u), & \text{if}\;u\in [0.65,0.70)\\ 0.0902617+u(-0.776093+(0.987053-0.299945u)u), & \text{if}\;u\in [0.70,0.75)\\ 0.1118810+u(-0.862570+(1.10236-0.351191u)u), & \text{if}\;u\in [0.75,0.80)\\ 0.1336910+u(-0.944358+(1.20459-0.393789u)u), & \text{if}\;u\in [0.80,0.85)\\ 0.1538970+u(-1.015670+(1.28849-0.426690u)u), & \text{if}\;u\in [0.85,0.90)\\ 0.1702230+u(-1.070090+(1.34896-0.449085u)u), & \text{if}\;u\in [0.90,0.95)\\ 0.1799430+u(-1.100790+(1.38127-0.460423u)u), & \text{if}\;u\in [0.95,1.00). \end{array} \end{array}$$ (37)

**Table 1:** For Example 6.1, absolute errors when *t* = 0.2, *N* = 500, *γ* = 1.8 and Δ*t* = 0.02.

u	Exact results	Approximate results	Absolute errors
0.1	−0.049442719100	−0.049442670935	4.81652 × 10^−8^
0.2	−0.094045640367	−0.094045548809	9.15580 × 10^−8^
0.3	−0.129442719100	−0.129442593109	1.25991 × 10^−7^
0.4	−0.152169042607	−0.152168894512	1.48096 × 10^−7^
0.5	−0.160000000000	−0.159999844291	1.55709 × 10^−7^
0.6	−0.152169042607	−0.152168894522	1.48085 × 10^−7^
0.7	−0.129442719100	−0.129442593133	1.25967 × 10^−7^
0.8	−0.094045640367	−0.094045548847	9.15200 × 10^−8^
0.9	−0.049442719100	−0.049442670985	4.81148 × 10^−8^

**Table 2:** Error norm with Δ*t* = 0.01 for Example 6.1 at different temporal values.

t	L_∞_		L_2_	
	*γ* = 1.9, *N* = 200	*γ* = 1.5, *N* = 450	*γ* = 1.9, *N* = 200	*γ* = 1.5, *N* = 450
0.2	7.49440 × 10^−7^	2.91633 × 10^−7^	5.29934 × 10^−7^	2.06207 × 10^−7^
0.4	3.33120 × 10^−6^	8.00613 × 10^−7^	2.35552 × 10^−6^	5.66104 × 10^−7^
0.6	6.48024 × 10^−6^	1.12398 × 10^−6^	4.58222 × 10^−6^	7.94759 × 10^−7^
0.8	7.78192 × 10^−6^	1.06115 × 10^−6^	5.50265 × 10^−6^	7.50338 × 10^−7^
1.0	5.41751 × 10^−6^	5.58223 × 10^−7^	3.83076 × 10^−6^	3.94722 × 10^−7^

**Table 3:** Error norm for Example 6.1 at various values of *h* when $\Delta t=\frac{1}{120}$, *t* = 1 and *γ* = 1.5.

N	L_∞_	L_2_	Order
10	0.001118246562	7.90720 × 10^−4^	⋯
20	2.82301 × 10^−4^	1.99617 × 10^−4^	1.985932
40	7.07469 × 10^−5^	5.00256 × 10^−5^	1.996495
80	1.76975 × 10^−5^	1.25140 × 10^−5^	1.999124
160	4.42504 × 10^−6^	3.12898 × 10^−6^	1.999781

**Table 4:** Error norm at *t* = 0.4 and *γ* = 1.7 for Example 6.1.

h	Δt	L_∞_	L_2_
$\frac{1}{10}$	$\frac{1}{50}$	0.001501405431	0.001061653961
$\frac{1}{20}$	$\frac{1}{100}$	3.79145 × 10^−4^	2.68096 × 10^−4^
$\frac{1}{30}$	$\frac{1}{200}$	1.69310 × 10^−4^	1.19720 × 10^−4^
$\frac{1}{50}$	$\frac{1}{250}$	6.10108 × 10^−5^	4.31411 × 10^−5^
$\frac{1}{50}$	$\frac{1}{300}$	6.10482 × 10^−5^	4.31676 × 10^−5^
$\frac{1}{60}$	$\frac{1}{400}$	4.24274 × 10^−5^	3.00007 × 10^−5^
$\frac{1}{60}$	$\frac{1}{450}$	4.24382 × 10^−5^	3.00083 × 10^−5^
$\frac{1}{75}$	$\frac{1}{480}$	2.71581 × 10^−5^	1.92079 × 10^−5^

**Table 5:** Error norms at distinct values of Δ*t* for Example 6.1 whereas *γ* = 1.5 and *t* = 1.

Δt	h = (Δt)^2^	L_∞_	L_2_
$\frac{1}{4}$	$\frac{1}{16}$	2.86977 × 10^−4^	2.02924 × 10^−4^
$\frac{1}{8}$	$\frac{1}{64}$	2.31102 × 10^−5^	1.63414 × 10^−5^
$\frac{1}{16}$	$\frac{1}{256}$	1.60317 × 10^−6^	1.13361 × 10^−6^
$\frac{1}{32}$	$\frac{1}{1024}$	1.04902 × 10^−7^	7.41782 × 10^−8^

**Example 6.2**. *Consider the TFDWE*

$$\frac{\partial^{\gamma }w\left(u,t\right)}{\partial t^{\gamma }}+\frac{\partial w(u,t)}{\partial t}-\frac{\partial^{2}w\left(u,t\right)}{\partial u^{2}}=q\left(u,t\right),\;\;\gamma \in \left(1,2\right),\;\;u\in \left[0,1\right],\;\;t\in \left[0,1\right],$$

*with ICs*

$$\begin{array}{c} w\left(u,0\right)=0,\;\;\;\;w_{t}\left(u,0\right)=0 \end{array}$$ (38)

*and the BCs*

$$\begin{array}{c} w(0,t)=0,\;\;\;\;w(1,t)=0, \end{array}$$ (39)

*where* $q\left(u,t\right)=2(\frac{R(\gamma )}{\gamma })u\left(1-u\right)(1-\;\text{exp}\;[-\frac{\gamma }{2-\gamma }t])+2tu\left(1-u\right)+2t^{2}$.

**Fig 4:**
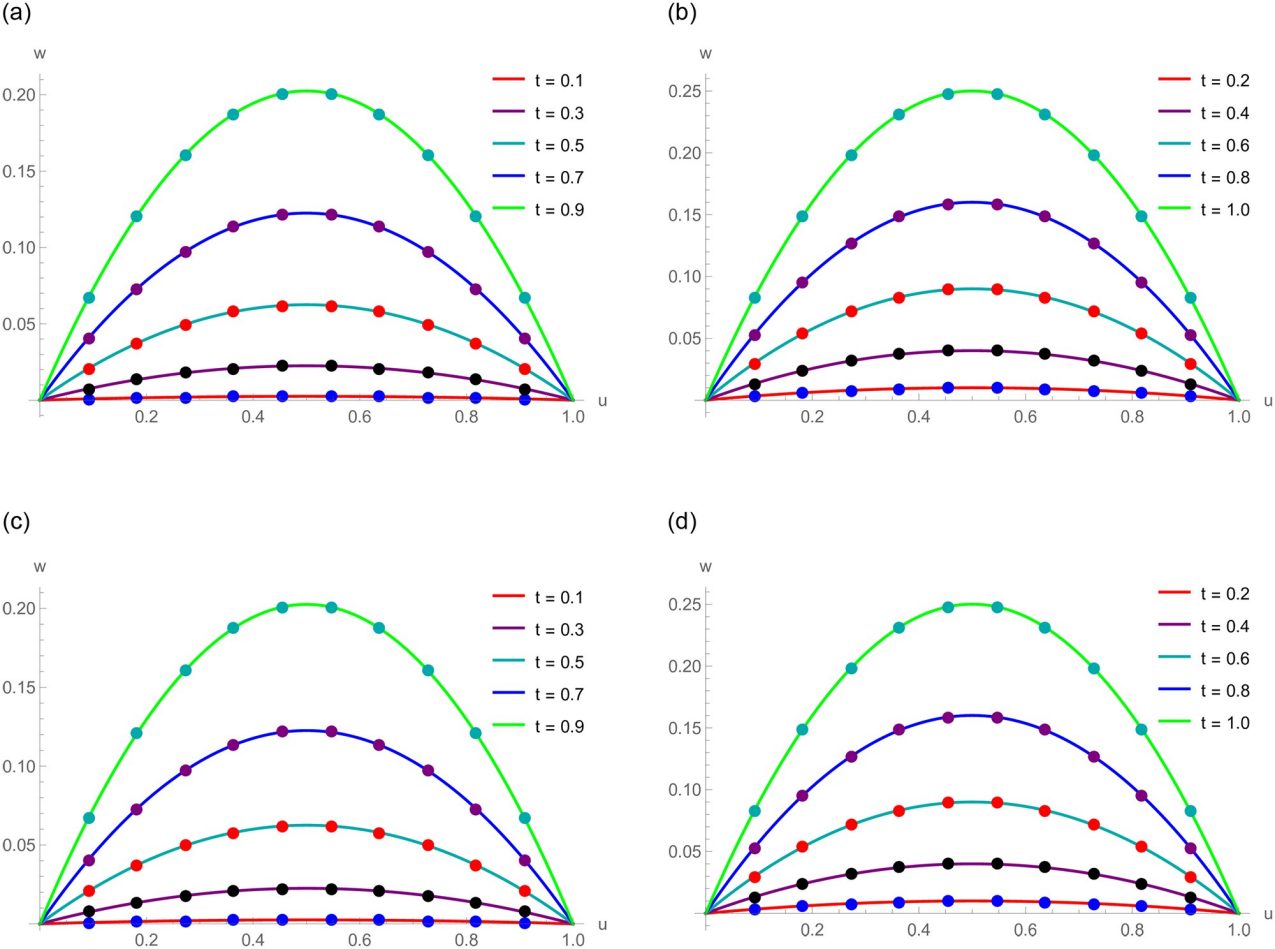
For Example 6.2, numerical and exact results at various temporal directions. (a) *N* = 120, *γ* = 1.4 and Δ*t* = 0.001 (b) *N* = 120, *γ* = 1.4 and Δ*t* = 0.001 (c) *N* = 90, *γ* = 1.7 and Δ*t* = 0.002 (d) *N* = 90, *γ* = 1.7 and Δ*t* = 0.002.

**Fig 5:**
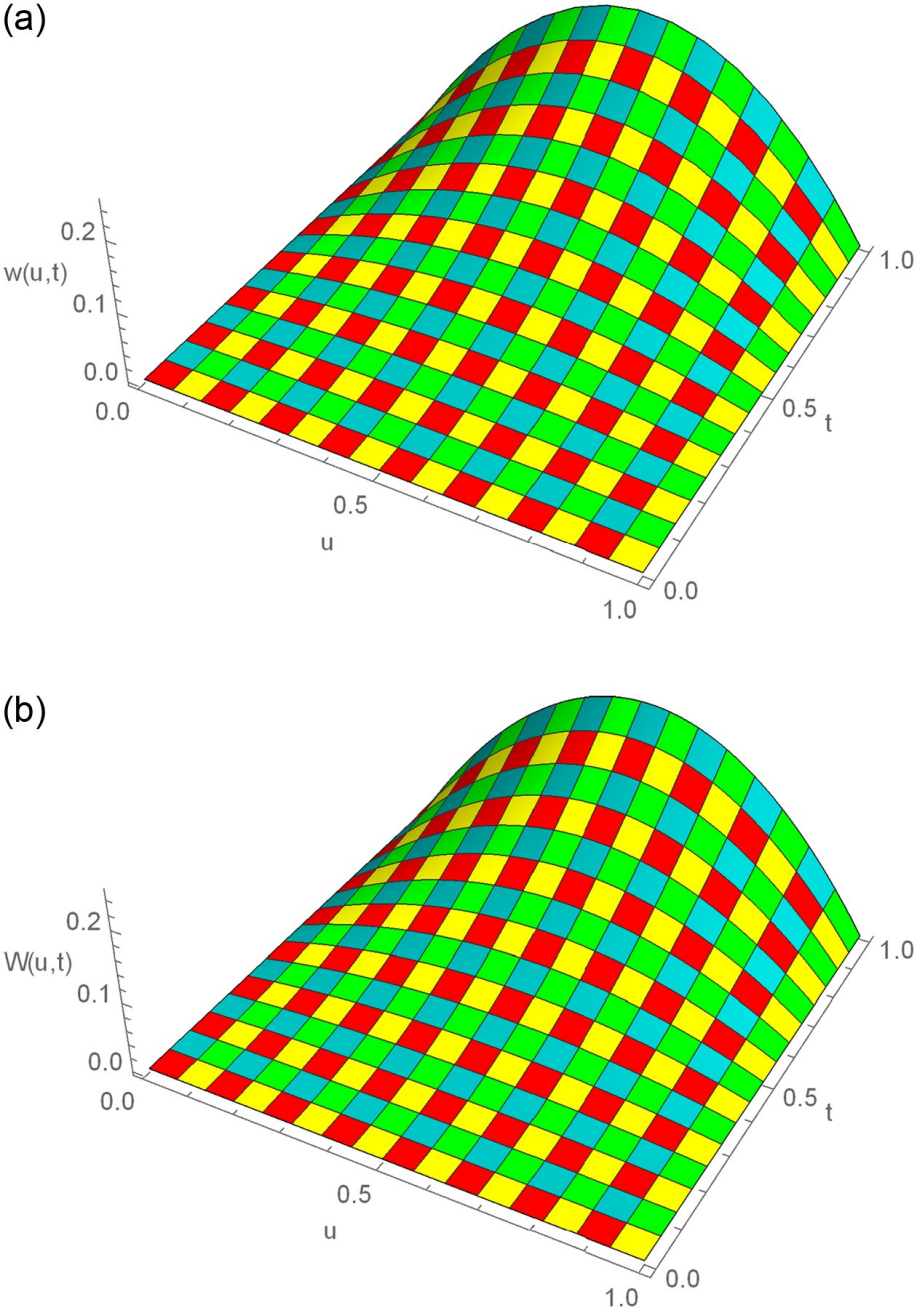
3D exact and numerical solutions for Example 6.2, where *t* = 1, *N* = 200, Δ*t* = 0.001, *γ* = 1.5 and *u* ∈ [0, 1]. (a) Exact result (b) Approximate result.

**Fig 6:**
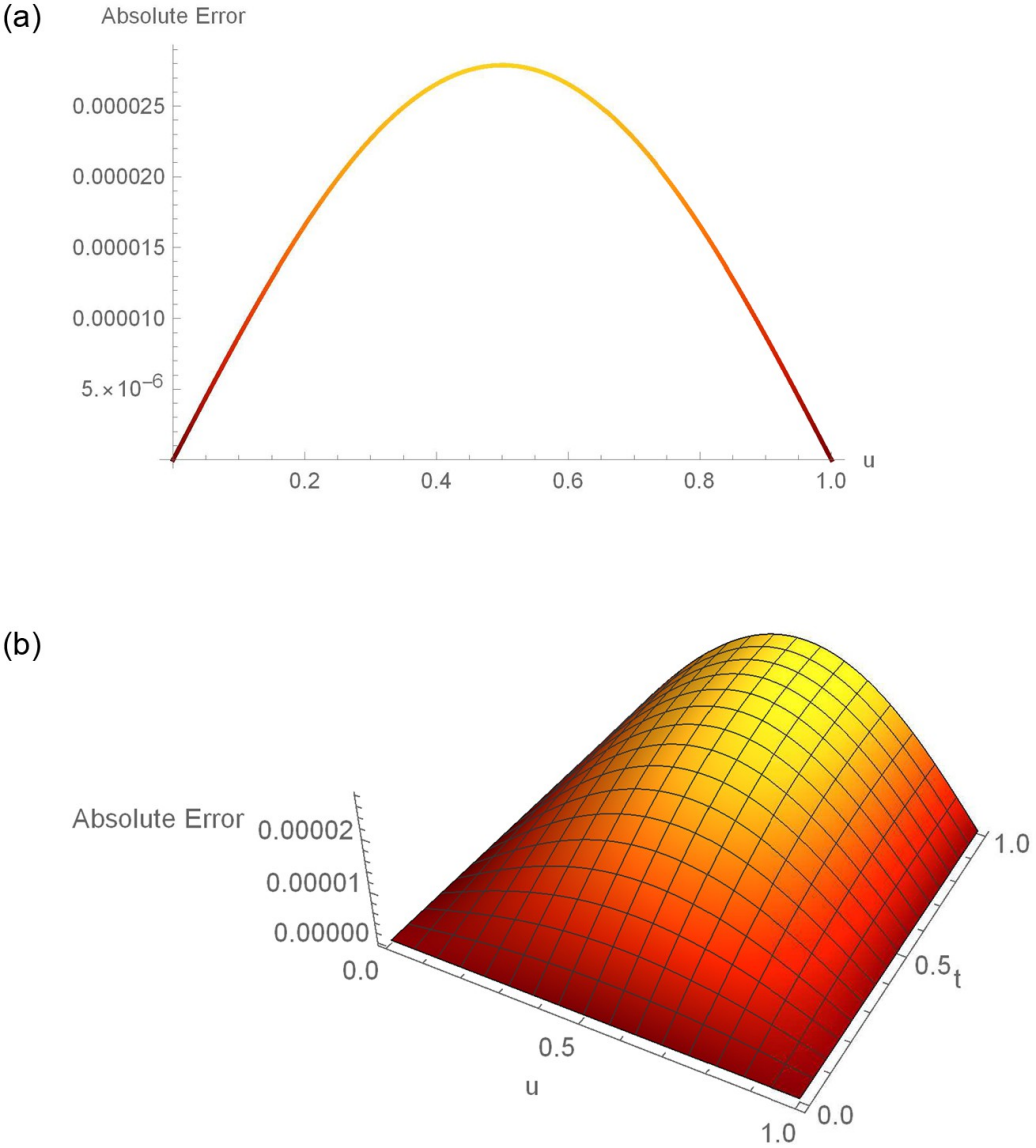
2D and 3D error profiles for Δ*t* = 0.001, *N* = 200,*γ* = 1.5, *u* ∈ [0, 1] and *t* = 1 of Example 6.2. (a) 2D error function (b) 3D error function.

The *w*(*u*, *t*) = *t*^2^*u*(1−*u*) is the exact solution. Table 6 displays the absolute error and the approximate results of Example 6.2 at various spatial grid values. At numerous time stages, error norms *L*_2_ and *L*_∞_ are represented in Table 7. Table 8 comprises the analysis of error norms and convergence order. Table 9 describes the error norm at several values of Δ*t*. Fig 4 exhibits the relation between analytical solutions and numerical outcomes at different temporal directions. 3D precision of the existing approach is displayed by graphs of numerical results and analytic solutions in Fig 5. Fig 6 demonstrates the 2D and 3D errors description. Tables and figures dipict that the presented numerical results are adaptable with exact solutions. The piecewise CBS numerical solutions when *γ* = 1.8, *N* = 50, Δ*t* = 0.01, *t* = 1 and *γ* = 1.6, *N* = 20, Δ*t* = 0.001, *t* = 0.5 of Example 6.2, are recorded in Eqs (40) and (41), respectively.

$$\begin{array}{c} W\left(u,1\right)=\{\begin{array}{cc} -6.97688\times 10^{-14}+u(1.001+(-1.00000-0.00191021u)u), & \text{if}\;u\in [0.00,0.02)\\ -5.42141\times 10^{-10}+u(1.001+(-1.00000-0.00184245u)u), & \text{if}\;u\in [0.02,0.04)\\ -4.94836\times 10^{-9}+u(1.001+(-1.00001-0.00177361u)u), & \text{if}\;u\in [0.04,0.06)\\ -2.00526\times 10^{-8}+u(1.001+(-1.00002-0.00170368u)u), & \text{if}\;u\in [0.06,0.08)\\ -5.64017\times 10^{-8}+u(1.001+(-1.00004-0.00163268u)u), & \text{if}\;u\in [0.08,0.10)\\ -1.28446\times 10^{-7}+u(1.00101+(-1.00006-0.00156064u)u), & \text{if}\;u\in [0.10,0.12)\\ -2.54716\times 10^{-7}+u(1.00101+(-1.00009-0.00148757u)u), & \text{if}\;u\in [0.12,0.14)\\ -4.57982\times 10^{-7}+u(1.00101+(-1.00012-0.00141349u)u), & \text{if}\;u\in [0.14,0.16)\\ \;\vdots \\ -4.19964\times 10^{-5}+u(1.00137+(-1.00121-0.000210002u)u), & \text{if}\;u\in [0.44,0.46)\\ -5.01661\times 10^{-5}+u(1.00142+(-1.00133-0.00012607u)u), & \text{if}\;u\in [0.46,0.48)\\ -5.94597\times 10^{-5}+u(1.00148+(-1.00145-0.0000420347u)u), & \text{if}\;u\in [0.48,0.50)\\ -6.99684\times 10^{-5}+u(1.00155+(-1.00158+0.0000420347u)u), & \text{if}\;u\in [0.50,0.52)\\ -8.17844\times 10^{-5}+u(1.00161+(-1.00171+0.00012607u)u), & \text{if}\;u\in [0.52,0.54)\\ \;\vdots \\ -0.000521351+u(1.00347+(-1.00436+0.00141349u)u), & \text{if}\;u\in [0.84,0.86)\\ -0.000568468+u(1.00363+(-1.00455+0.00148757u)u), & \text{if}\;u\in [0.86,0.88)\\ -0.000618265+u(1.00380+(-1.00475+0.00156064u)u), & \text{if}\;u\in [0.88,0.90)\\ -0.000670785+u(1.00398+(-1.00494+0.00163268u)u), & \text{if}\;u\in [0.90,0.92)\\ -0.000726068+u(1.00416+(-1.00514+0.00170368u)u), & \text{if}\;u\in [0.92,0.94)\\ -0.000784148+u(1.00434+(-1.00533+0.00177361u)u), & \text{if}\;u\in [0.94,0.96)\\ -0.000845059+u(1.00453+(-1.00553+0.00184245u)u), & \text{if}\;u\in [0.96,0.98)\\ -0.000908833+u(1.00473+(-1.00573+0.00191021u)u), & \text{if}\;u\in [0.98,1.00). \end{array} \end{array}$$ (40)

and

$$\begin{array}{c} W\left(u,0.5\right)=\{\begin{array}{cc} -1.84922\times 10^{-15}+u(0.250073+(-0.250000-0.000140563u)u), & \text{if}\;u\in [0.00,0.05)\\ -1.95637\times 10^{-9}+u(0.250073+(-0.250002-0.000124912u)u), & \text{if}\;u\in [0.05,0.10)\\ -1.73370\times 10^{-8}+u(0.250073+(-0.250007-0.000109532u)u), & \text{if}\;u\in [0.10,0.15)\\ -6.86002\times 10^{-8}+u(0.250074+(-0.250014-0.0000943424u)u), & \text{if}\;u\in [0.15,0.20)\\ -1.88405\times 10^{-7}+u(0.250076+(-0.250023-0.0000793668u)u), & \text{if}\;u\in [0.20,0.25)\\ -4.19156\times 10^{-7}+u(0.250079+(-0.250034-0.0000645987u)u), & \text{if}\;u\in [0.25,0.30)\\ -8.12752\times 10^{-7}+u(0.250083+(-0.250047-0.0000500211u)u), & \text{if}\;u\in [0.30,0.35)\\ -1.43084\times 10^{-6}+u(0.250088+(-0.250062-0.000035605u)u), & \text{if}\;u\in [0.35,0.40)\\ -2.34561\times 10^{-6}+u(0.250095+(-0.250079-0.0000213117u)u), & \text{if}\;u\in [0.40,0.45)\\ -3.64109\times 10^{-6}+u(0.250104+(-0.250098-7.09520\times 10^{-6}u)u), & \text{if}\;u\in [0.45,0.50)\\ -5.41489\times 10^{-6}+u(0.250114+(-0.250120+7.09520\times 10^{-6}u)u), & \text{if}\;u\in [0.50,0.55)\\ -7.78017\times 10^{-6}+u(0.250127+(-0.250143+0.0000213117u)u), & \text{if}\;u\in [0.55,0.60)\\ -1.08675\times 10^{-5}+u(0.250143+(-0.250169+0.000035605u)u), & \text{if}\;u\in [0.60,0.65)\\ -1.48265\times 10^{-5}+u(0.250161+(-0.250197+0.0000500211u)u), & \text{if}\;u\in [0.65,0.70)\\ -1.98267\times 10^{-5}+u(0.250182+(-0.250228+0.0000645987u)u), & \text{if}\;u\in [0.70,0.75)\\ -2.60569\times 10^{-5}+u(0.250207+(-0.250261+0.0000793668u)u), & \text{if}\;u\in [0.75,0.80)\\ -3.37245\times 10^{-5}+u(0.250236+(-0.250297+0.0000943424u)u), & \text{if}\;u\in [0.80,0.85)\\ -4.30525\times 10^{-5}+u(0.250269+(-0.250336+0.000109532u)u), & \text{if}\;u\in [0.85,0.90)\\ -5.42649\times 10^{-5}+u(0.250306+(-0.250377+0.000124912u)u), & \text{if}\;u\in [0.90,0.95)\\ -6.76837\times 10^{-5}+u(0.250349+(-0.250422+0.000140563u)u), & \text{if}\;u\in [0.95,1.00). \end{array} \end{array}$$ (41)

**Table 6:** Absolute errors for Example 6.2 whereas *t* = 0.1, *γ* = 1.9, *N* = 10 and Δ*t* = 0.0001.

u	Exact results	Approximate results	Absolute errors
0.1	0.000900000000	0.000900101445	1.01445 × 10^−07^
0.2	0.001600000000	0.001600187058	1.87058 × 10^−07^
0.3	0.002100000000	0.002100249576	2.49576 × 10^−07^
0.4	0.002400000000	0.002400287195	2.87195 × 10^−07^
0.5	0.002500000000	0.002500299728	2.99728 × 10^−07^
0.6	0.002400000000	0.002400287195	2.87195 × 10^−07^
0.7	0.002100000000	0.002100249576	2.49576 × 10^−07^
0.8	0.001600000000	0.001600187058	1.87058 × 10^−07^
0.9	0.000900000000	0.000900101445	1.01445 × 10^−07^

**Table 7:** Error norms with Δ*t* = 0.001 and *N* = 10 for Example 6.2 at various time stages.

t	L_∞_		L_2_	
	*γ* = 1.9	*γ* = 1.4	*γ* = 1.9	*γ* = 1.4
0.2	8.69048 × 10^−6^	1.05677 × 10^−5^	6.22793 × 10^−6^	7.52361 × 10^−6^
0.4	2.25387 × 10^−5^	1.81730 × 10^−5^	1.60045 × 10^−5^	1.29073 × 10^−5^
0.6	3.23597 × 10^−5^	2.28951 × 10^−5^	2.29458 × 10^−5^	1.62500 × 10^−5^
0.8	3.48563 × 10^−5^	2.54256 × 10^−5^	2.47146 × 10^−5^	1.80415 × 10^−5^
1.0	3.17836 × 10^−5^	2.65240 × 10^−5^	2.25406 × 10^−5^	1.88195 × 10^−5^

**Table 8:** Error norms at various values of *h* for Example 6.2 whereas *γ* = 1.5 and *t* = 1.

h	Δt = h^2^	L_∞_	L_2_	Order
$\frac{1}{4}$	$\frac{1}{16}$	0.001633418622	0.001157633552	⋯
$\frac{1}{8}$	$\frac{1}{64}$	4.28928 × 10^−4^	3.04282 × 10^−4^	1.929088
$\frac{1}{16}$	$\frac{1}{256}$	1.08548 × 10^−4^	7.70216 × 10^−5^	1.982408
$\frac{1}{32}$	$\frac{1}{1024}$	2.72196 × 10^−5^	1.93152 × 10^−5^	1.995609

**Table 9:** Error norms at various values of $\Delta t=\frac{1}{M}$ when *N* = 10, *t* = 1 and *γ* = 1.3 of Example 6.2.

M	L_∞_	L_2_
10	0.002533892437	0.001798283755
20	0.001271777484	9.02525 × 10^−4^
40	6.36685 × 10^−4^	4.51809 × 10^−4^
80	3.18486 × 10^−4^	2.25999 × 10^−4^
160	1.59272 × 10^−4^	1.13018 × 10^−4^

**Example 6.3**. *Consider the TFDWE*

$$\frac{\partial^{\gamma }w\left(u,t\right)}{\partial t^{\gamma }}+w\left(u,t\right)-\frac{\partial^{2}w\left(u,t\right)}{\partial u^{2}}=q\left(u,t\right),\;\;\gamma \in \left(1,2\right),\;\;u\in \left[0,1\right],\;\;t\in \left[0,1\right],$$

*with ICs*

$$\begin{array}{c} w\left(u,0\right)=0,\;\;\;\;w_{t}\left(u,0\right)=0 \end{array}$$ (42)

*and the BCs*

$$\begin{array}{c} w\left(0,t\right)=0,\;\;\;\;w\left(1,t\right)=t^{2}\text{sinh}\left(1\right), \end{array}$$ (43)

*where* $q\left(u,t\right)=2(\frac{R(\gamma )}{\gamma })\text{sinh}\left(u\right)(1-\;\text{exp}\;[-\frac{\gamma }{2-\gamma }t])$.

**Fig 7:**
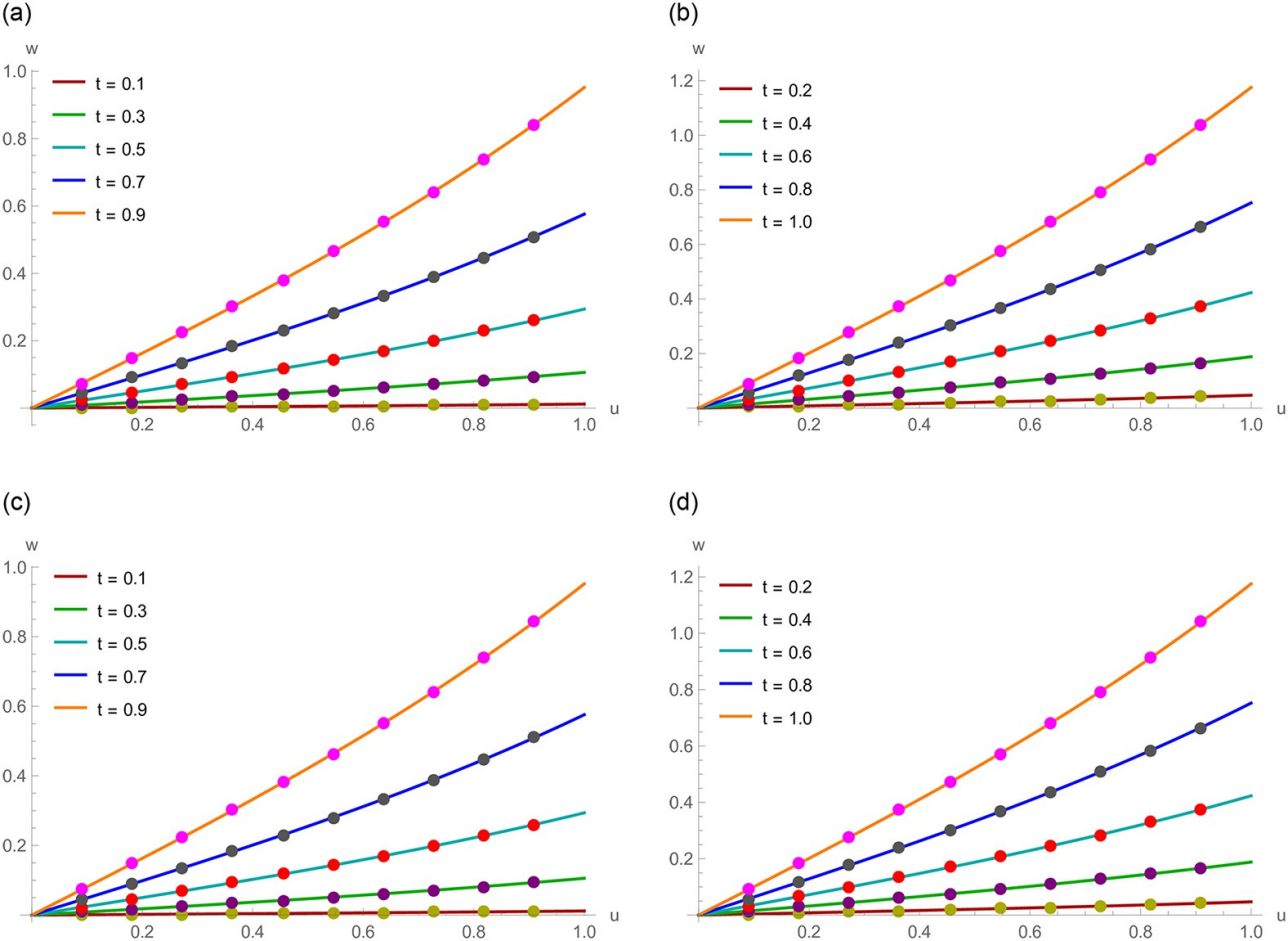
For Example 6.3, exact and numerical results at various levels of time. (a) *N* = 80, *γ* = 1.3 and Δ*t* = 0.005 (b) *N* = 80, *γ* = 1.3 and Δ*t* = 0.005 (c) *N* = 450, *γ* = 1.6 and Δ*t* = 0.01 (d) *N* = 450, *γ* = 1.6 and Δ*t* = 0.01.

**Fig 8:**
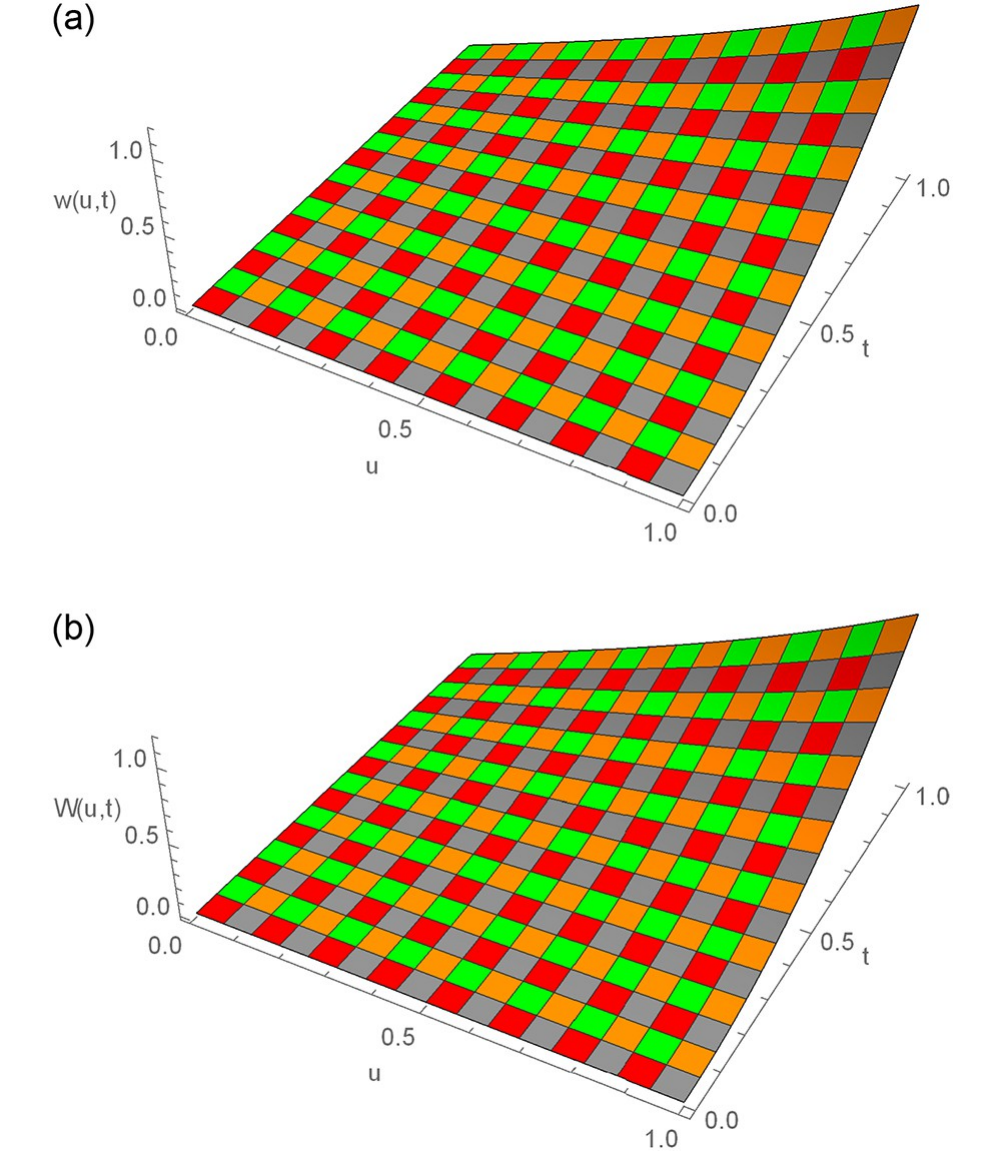
3D exact and numerical solutions for Example 6.3, where *t* = 1, *N* = 150, Δ*t* = 0.01, *γ* = 1.5 and *u* ∈ [0, 1]. (a) Exact result (b) Approximate result.

**Fig 9:**
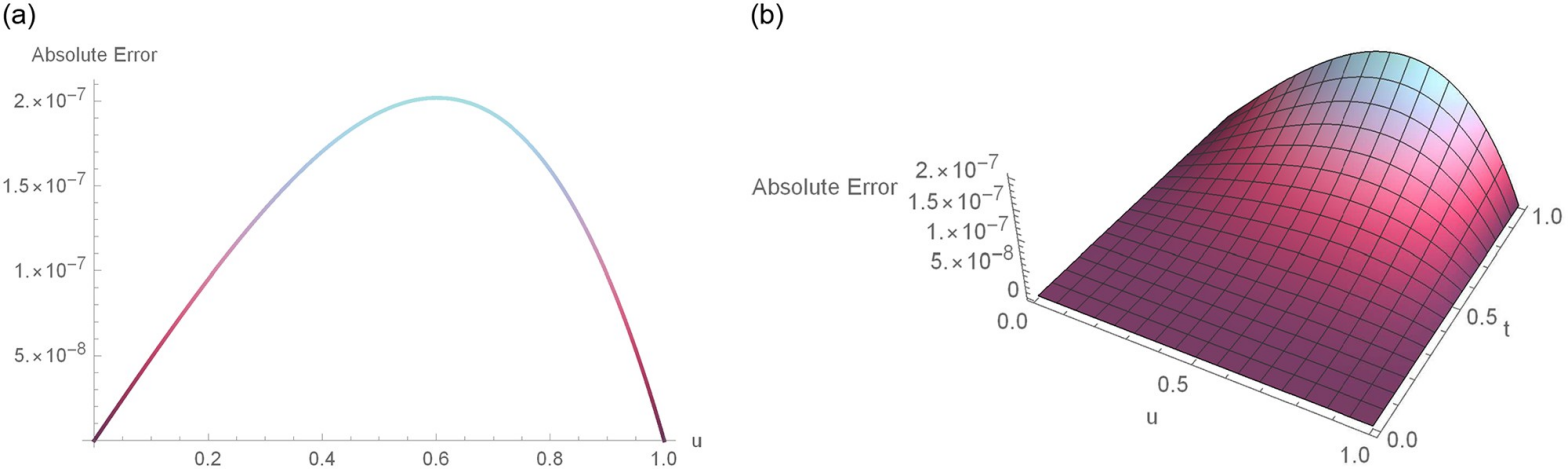
2D and 3D error profiles for Δ*t* = 0.01, *N* = 150,*γ* = 1.5, *u* ∈ [0, 1] and *t* = 1 for Example 6.3. (a) 2D error function (b) 3D error function.

The *w*(*u*, *t*) = *t*^2^sinh(*u*) is the exact solution. Table 10 displays computational outcomes and absolute error of Example 6.3 at various spatial grid values. Table 11 expounds the analysis of error norms and convergence order in the spatial direction. Table 12 describes the error norm at several values of Δ*t*. Table 13 depicts the error norm for *γ* = 1.5, 1.9 at numerous time stages. Fig 7 exhibits the relation between analytical results and numerical outcomes at different temporal directions. 3D precision of the existing approach is displayed by graphs of numerical results and analytic solutions in Fig 8. Fig 9 demonstrates the 2D and 3D errors description. The piecewise CBS numerical solutions when *γ* = 1.4, *N* = 20, Δ*t* = 0.01, *t* = 1 and *γ* = 1.8, *N* = 50, Δ*t* = 0.001, *t* = 0.5 of Example 6.3, are written in Eqs (44) and (45), respectively.

$$\begin{array}{c} W\left(u,1\right)=\{\begin{array}{cc} 3.91596\times 10^{-14}+u(0.999972+(3.28093\times 10^{-12}+0.166724u)u), & \text{if}\;u\in [0.00,0.05)\\ -5.21364\times 10^{-8}+u(0.999975+(-0.0000625637+0.167141u)u), & \text{if}\;u\in [0.05,0.10)\\ -8.87363\times 10^{-7}+u(1.00000+(-0.000313132+0.167976u)u), & \text{if}\;u\in [0.10,0.15)\\ -5.12451\times 10^{-6}+u(1.00009+(-0.000878085+0.169232u)u), & \text{if}\;u\in [0.15,0.20)\\ -1.8555\times 10^{-5}+u(1.00029+(-0.00188537+0.17091u)u), & \text{if}\;u\in [0.20,0.25)\\ -5.14673\times 10^{-5}+u(1.00068+(-0.00346516+0.173017u)u), & \text{if}\;u\in [0.25,0.30)\\ -0.000120026+u(1.00137+(-0.00575046+0.175556u)u), & \text{if}\;u\in [0.30,0.35)\\ -0.000247725+u(1.00246+(-0.00887778+0.178534u)u), & \text{if}\;u\in [0.35,0.40)\\ -0.000466928+u(1.00411+(-0.0129878+0.18196u)u), & \text{if}\;u\in [0.40,0.45)\\ -0.000820516+u(1.00646+(-0.0182262+0.18584u)u), & \text{if}\;u\in [0.45,0.50)\\ -0.00136366+u(1.00972+(-0.0247439+0.190185u)u), & \text{if}\;u\in [0.50,0.55)\\ -0.00216575+u(1.01410+(-0.0326985+0.195006u)u), & \text{if}\;u\in [0.55,0.60)\\ -0.00331245+u(1.01983+(-0.0422543+0.200315u)u), & \text{if}\;u\in [0.60,0.65)\\ -0.004908+u(1.02719+(-0.0535837+0.206125u)u), & \text{if}\;u\in [0.65,0.70)\\ -0.00707768+u(1.03649+(-0.0668675+0.21245u)u), & \text{if}\;u\in [0.70,0.75)\\ -0.00997052+u(1.04806+(-0.0822959+0.219307u)u), & \text{if}\;u\in [0.75,0.80)\\ -0.0137623+u(1.06228+(-0.10007+0.226713u)u), & \text{if}\;u\in [0.80,0.85)\\ -0.0186586+u(1.07956+(-0.120401+0.234686u)u), & \text{if}\;u\in [0.85,0.90)\\ -0.0248989+u(1.10037+(-0.143513+0.243246u)u), & \text{if}\;u\in [0.90,0.95)\\ -0.0327598+u(1.12519+(-0.169643+0.252415u)u), & \text{if}\;u\in [0.95,1.00). \end{array} \end{array}$$ (44)

and

$$\begin{array}{c} W\left(u,0.5\right)=\{\begin{array}{cc} -2.68427\times 10^{-15}+u(0.249999+(-2.31939\times 10^{-11}+0.0416682u)u), & \text{if}\;u\in [0.00,0.02)\\ -1.33365\times 10^{-10}+u(0.249999+(-1.00024\times 10^{-6}+0.0416849u)u), & \text{if}\;u\in [0.02,0.04)\\ -2.26763\times 10^{-9}+u(0.250000+(-5.00199\times 10^{-6}+0.0417182u)u), & \text{if}\;u\in [0.04,0.06)\\ -1.30760\times 10^{-8}+u(0.250000+(-0.0000140089+0.0417683u)u), & \text{if}\;u\in [0.06,0.08)\\ -4.72516\times 10^{-8}+u(0.250001+(-0.0000300288+0.0418350u)u), & \text{if}\;u\in [0.08,0.10)\\ -1.30738\times 10^{-7}+u(0.250004+(-0.0000550748+0.0419185u)u), & \text{if}\;u\in [0.10,0.12)\\ -3.03984\times 10^{-7}+u(0.250008+(-0.0000911676+0.0420188u)u), & \text{if}\;u\in [0.12,0.14)\\ -6.25220\times 10^{-7}+u(0.250015+(-0.0001403360+0.0421358u)u), & \text{if}\;u\in [0.14,0.16)\\ \;\vdots \\ -0.000157275+u(0.251309+(-0.00387306+0.0459573u)u), & \text{if}\;u\in [0.44,0.46)\\ -0.000195925+u(0.251561+(-0.00442103+0.0463544u)u), & \text{if}\;u\in [0.46,0.48)\\ -0.000241889+u(0.251848+(-0.00501953+0.0467700u)u), & \text{if}\;u\in [0.48,0.50)\\ -0.000296181+u(0.252174+(-0.00567103+0.0472043u)u), & \text{if}\;u\in [0.50,0.52)\\ -0.000359908+u(0.252542+(-0.00637806+0.0476575u)u), & \text{if}\;u\in [0.52,0.54)\\ \;\vdots \\ -0.00402348+u(0.267656+(-0.0274870+0.0576496u)u), & \text{if}\;u\in [0.84,0.86)\\ -0.00453773+u(0.269450+(-0.0295730+0.0584581u)u), & \text{if}\;u\in [0.86,0.88)\\ -0.00510464+u(0.271383+(-0.0317692+0.059290u)u), & \text{if}\;u\in [0.88,0.90)\\ -0.00572839+u(0.273462+(-0.0340794+0.0601456u)u), & \text{if}\;u\in [0.90,0.92)\\ -0.00641338+u(0.275695+(-0.0365073+0.0610253u)u), & \text{if}\;u\in [0.92,0.94)\\ -0.00716431+u(0.278092+(-0.0390568+0.0619294u)u), & \text{if}\;u\in [0.94,0.96)\\ -0.00798611+u(0.280660+(-0.0417319+0.0628583u)u), & \text{if}\;u\in [0.96,0.98)\\ -0.00888402+u(0.283409+(-0.0445367+0.0638123u)u), & \text{if}\;u\in [0.98,1.00). \end{array} \end{array}$$ (45)

**Table 10:** For Example 6.3, absolute errors whereas *t* = 1, *N* = 200, *γ* = 1.25 and Δ*t* = 0.01.

u	Exact results	Approximate results	Absolute errors
0.1	0.100166750020	0.100166722376	2.76439 × 10^−8^
0.2	0.201336002541	0.201335948621	5.39201 × 10^−8^
0.3	0.304520293447	0.304520216068	7.73796 × 10^−8^
0.4	0.410752325803	0.410752229339	9.64634 × 10^−8^
0.5	0.521095305494	0.521095196063	1.09431 × 10^−7^
0.6	0.636653582148	0.636653467855	1.14294 × 10^−7^
0.7	0.758583701840	0.758583593098	1.08742 × 10^−7^
0.8	0.888105982188	0.888105892120	9.00675 × 10^−8^
0.9	1.026516725708	1.026516670630	5.50781 × 10^−8^

**Table 11:** Error norms for Example 6.3 at numerous values of *h* when *γ* = 1.7 and *t* = 1.

h	Δt = h^2^	L_∞_	L_2_	Order
$\frac{1}{4}$	$\frac{1}{16}$	2.76259 × 10^−4^	2.05926 × 10^−4^	⋯
$\frac{1}{8}$	$\frac{1}{64}$	7.11274 × 10^−5^	5.10360 × 10^−5^	1.957544
$\frac{1}{16}$	$\frac{1}{256}$	1.77292 × 10^−5^	1.27239 × 10^−5^	2.004280
$\frac{1}{32}$	$\frac{1}{1024}$	4.43898 × 10^−6^	3.17867 × 10^−6^	1.997826

**Table 12:** Error norms at various values of Δ*t* when *t* = 1 and *γ* = 1.6 of Example 6.3.

Δt	h = (Δt)^2^	L_∞_	L_2_
$\frac{1}{4}$	$\frac{1}{16}$	1.78317 × 10^−5^	1.28034 × 10^−5^
$\frac{1}{8}$	$\frac{1}{64}$	1.11080 × 10^−6^	7.95517 × 10^−7^
$\frac{1}{16}$	$\frac{1}{256}$	6.93159 × 10^−8^	4.96288 × 10^−8^
$\frac{1}{32}$	$\frac{1}{1024}$	5.25581 × 10^−9^	4.31729 × 10^−9^

**Table 13:** Error norm with Δ*t* = 0.01 and *N* = 500 for Example 6.3 at distinct time stages.

t	L_∞_		L_2_	
	*γ* = 1.5	*γ* = 1.9	*γ* = 1.5	*γ* = 1.9
0.2	2.96264 × 10^−10^	1.40314 × 10^−10^	2.06557 × 10^−10^	9.17470 × 10^−11^
0.4	1.90131 × 10^−09^	1.26824 × 10^−09^	1.35281 × 10^−09^	8.67226 × 10^−10^
0.6	5.38140 × 10^−09^	4.43279 × 10^−09^	3.85198 × 10^−09^	3.11207 × 10^−09^
0.8	1.08568 × 10^−08^	1.01962 × 10^−08^	7.78694 × 10^−09^	7.25512 × 10^−09^
1.0	1.82507 × 10^−08^	1.84028 × 10^−08^	1.30966 × 10^−08^	1.31575 × 10^−08^

## 7 Conclusion

In this paper, we addressed the problem of finding numerical solutions to time FPDE. For this purpose, B-splines were utilized to build up a collocation technique for TFDWE. The procedure employed the typical FDM to approximate the time fractional derivative, whereas the derivative in space was discretized utilizing the cubic B-splines. Further, the effective numerical scheme to examine the numerical solutions of TFDWE involving damping and reaction terms has been presented. We have used the CBS functions and *θ*-weighted scheme with CFFD. The proposed scheme is stable unconditionally possessing second order temporal and spatial convergence. Three numerical problems have been analyzed. Numerical and graphical comparison uncovers that the provided method is accurate and computationally very effective. In future, we may consider the higher dimensional and variable order FPDEs for numerical solutions via spline functions.
